# Supplementary material for: A comparison of two manufacturing methods in the phase I COBALT study of CD19CAR T for LBCL
Source: Mol Ther Adv. 2026 Feb 13;34(1):201695. doi: 10.1016/j.omta.2026.201695 (PMC13148904; doi:10.1016/j.omta.2026.201695)
Supplement: Document S1. Figures S1–S6 and Tables S1–S7 [file mmc1.pdf]

OMTA, Volume *34*

Supplemental information

A comparison of two manufacturing methods in the phase I COBALT study of CD19CAR T for LBCL

Claire Roddie, Juliana Dias, Gordon Weng-Kit Cheung, Maeve A. O'Reilly, Mahnaz Abbasian, Amaia Cadinanos-Garai, Ketki Vispute, Leticia Bosshard-Carter, Marina Mitsikakou, Vedika Mehra, Harriet Roddy, John A. Hartley, Nasir G. Mahmoud, Leah Ensell, Yashma Patel, Maria A.V. Marzolini, Farzin Farzaneh, Nourredine Himoudi, Farhatullah Syed, Bilyana Popova, Andre Lopes, Alexander Day, Mark W. Lowdell, and Karl S. Peggs

SUPPLEMENTAL TABLES:

Table S1: COBALT inclusion and exclusion criteria

**Inclusion**
• Age 16-65 years<br>• Confirmed diagnosis of CD19+ DLBCL<br>• Primary resistant or relapsed disease failing to achieve metabolic CR to 1st line salvage, or relapse post autograft failing to achieve metabolic CR following a single further cycle of salvage<br>• Potential allogeneic transplant candidate<br>• Agreement to have a pregnancy test, use adequate contraception for 12 months post 4G7CAR-T cell infusion<br>• Karnofsky performance status >60<br>• Written informed consent
**Exclusion at Registration**
• Women who are pregnant or lactating<br>• Prior allogeneic transplantation<br>• Prior history of ischaemic heart disease, dysrhythmias, abnormal ECG, MUGA LVEF<40% (if performed)<br>• Exclusions for proceeding to allogeneic transplantation (active HBV, HCV, HIV; LFT >3 x ULN; CrCl <40 ml/min; or other comorbidity that precludes transplantation)<br>• Known CNS involvement or CVA within prior 3 months<br>• Corticosteroids at a dose of >10mg prednisolone per day (or equivalent)<br>• Rituximab within the last 2 months prior to 4G7CAR-T infusion<br>• Active autoimmune disease requiring immunosuppression<br>• Life expectancy <3 months<br>• Known allergy to albumin or DMSO<br>• Any contraindication to the administration and use of ifosfamide, epirubicin, etoposide, fludarabine and cyclophosphamide.
**Exclusion for CD19CAR T-cell infusion at Day 0**
• Severe intercurrent infection at the time of scheduled 4G7CAR-T cell infusion<br>• Requirement for supplementary oxygen or active pulmonary infiltrates at the time of scheduled 4G7CAR-T cell infusion

Table S2: COBALT primary and secondary endpoints

**Primary endpoints**	**Secondary endpoints**
• Toxicity evaluation following 4G7CAR-T cell administration - most notably attributable AEs and SAEs<br>• Complete tumour response<br>• Feasibility of adequate leukapheresis collection and generation of 4G7CAR-T cells	• Engraftment, expansion and persistence of 4G7CAR-T cells<br>• Depletion of B cell compartment<br>• Timing and magnitude of cytokine release<br>• PET-CT response at 28 days<br>• Number of patients proceeding to allogeneic transplantation

Table S3: COBALT 4G7CAR-T product release criteria

Test/Facility	*Method*	*Specification*
**Cell Count**	FACS: BD Tru- Count™ absolute counting tubes 7AAD, CD45+CD3+ Manual cell count after trypan blue staining	Minimum cell dose<br>1 x 105/kg 4G7CAR-T cells
**Viability of cell product**	BD TruCount™/ Flow Cytometry for CD45, CD3 and 7AAD	≥70% viability (Pre-cryopreservation)
**Transduction efficiency**	Flow Cytometry	≥10% CAR+ cells (Live CD3+/CD45+ T cells)
**Sterility (Bacterial/ Fungal)**	Bacterial cultures	No Growth
**Mycoplasma**	PCR for 16S rRNA	None detected
**Endotoxin**	Kinetic Turbidimetric	≤2EU/ml
**Residual beads**	Manual count	<1bead per 1 x 10^6 cells

Table S4: Detailed demographic information for all registered patients on the COBALT study**.** Key: LD= lymphodepletion; IPI= International Prognostic Index; M/F= male/female; CMR= complete metabolic response; PD= progressive disease; PR= partial response; SD= stable disease; Leuk= leukapheresis (this material was used for head-to-head validations); X, Y, Z are patients with products manufactured on Process-A for the study whose surplus cryopreserved leukapheresis material was used for Process-B validations (see Table 1, main manuscript); *= Product did not meet target dose.

Age (y)	*M/F*	*Disease*	*IPI*	*Stage pre-LD*	*EN sites*	*Prior Therapy*	*Prior lines*	*Primary refractory*	*Bridging*	*LDH (IU/mL)*
*59	M	DLBCL (GC)	3	4a	Mesentery	CHOP-R x 5 to PD<br>ICE x 2 to PD	3	Yes	Pixantrone x1	481
52 Leuk X	F	tFL (GC)	2	3a	nil	R-CVP x 3 to PR (FL)<br>R-Benda x 6 to CMR + maintenance R (FL)<br>IVE x 2 to SD (tFL)<br>R-GDP x 2 to PD (tFL)<br>Pixantrone to SD (tFL)	5	Yes (once tFL)	Nil	271
55	M	tFL (GC)	1	2a	nil	R-CHOP x 6 + R x 2 to PD<br>R-GDP x 2 to PD	3	Yes	IVE x 1	289
*50 Leuk Y	M	DLBCL (non-GC)	3	4b	Liver	R-CHOP x 8 to PD<br>R-GDP x 1 to PD<br>R-IVE x 1 to PD<br>BTKi + Idelalasib (trial)-stopped due to toxicity<br>Lenalidomide to PD<br>Pixantrone x 1 to PD + mediastinal RT	6	Yes	Nil	292
*52 Leuk Z	M	tFL (GC)	3	4a	Pancreas	R-CHOP x 6 to CMR<br>R-DHAP x 2 to PD<br>IVE x 2 to PR	3	No	Tonsillar RT 30Gy in 15#	317
51	M	CD30+ tFL (GC)	4	4a	Bone marrow	ABVD x 2 to PR (PET+)<br>escBeacopp x 4 to CMR<br>ESHAP x 2 to CMR + LEAM ASCT<br>Brentuximab x 3 to mixed response<br>Nivolumab x 8 to PD	5	No	Steroids	624
63	F	DLBCL (GC)	4	4a	Lung	R-CHOP x 6 (+ HD MTX; IT MTX) to PR + RT to CMR<br>R-GDP x 4 –to mixed response<br>MiniLEAM x 1 to PD<br>Pixantrone x 4 to SD	6	No	IVE x 1	470
44	M	tFL (GC)	3	4a	Pleura	R-CHOP x 6 to CR + maintenance R<br>R-GDP x 2 (complicated by deafness) to PD<br>IVE x 1 to PD	4	Yes	mini-Leam x 1	283
48	M	tFL (GC)	3	4a	Bone	R-CHOP x 6 to PR + IFRT to tonsil to PR<br>R-GDP x 2 to PR<br>IVE x 1 to PR<br>Flu/Cy + Tisagenlecleucel to PD	4	Yes	Steroids	407
*64	F	DLBCL (non-GC)	3	2bx	nil	R-CHOP x 6 to PR<br>R-GDP x 2 to PMR + 30Gy RT in 15# to CMR<br>Flu/Cy + Tisagenlecleucel to PD	4	No	Steroids	537

						RBP x 3 to PR (stopped – cytopenias + infection)				

Table S5: COBALT 4G7CAR-T product details, according to release criteria.

Process	*Fresh/ Frozen Starting Material*	*Target Dose Level (DL)*	*CAR % of CD3*	*CD3 viability %*	*Total CAR T-cell yield ($x10^6$)*	*Target Dose Met (Yes/No) cryopreserved dose ($x10^6$)*	*Sterility (no growth 10d)*	*Endotoxin*	*Mycoplasma*
A	Fresh	DL1 2×10^5/kg	36.8	95.1	15.8	No (12)	No growth	≤2EU/ml	None detected
A	Fresh	DL1 2×10^5/kg	18.1	94.5	13.6	Yes (12.6)	No growth	≤2EU/ml	None detected
A	Fresh	DL1 2×10^5/kg	16	94	56.2	Yes (19)	No growth	≤2EU/ml	None detected
A	Fresh	DL2 1×10^6/kg	13.7	95	39.7	No (19.8)	No growth	≤2EU/ml	None detected
A	Fresh	DL2 1×10^6/kg	15.3	97	64.9	No (58)	No growth	≤2EU/ml	None detected
B	Frozen	DL2 1×10^6/kg	35.4	99.7	792	Yes(75.4)	No growth	≤2EU/ml	None detected
B	Fresh	DL2 1×10^6/kg	27.9	99.2	529	Yes (91.9)	No growth	≤2EU/ml	None detected
B	Frozen	DL2 1×10^6/kg	31.5	99.6	546	Yes (85.5)	No growth	≤2EU/ml	None detected
B	Fresh	DL3 5×10^6/kg	19.9	99.5	548	Yes (492)	No growth	≤2EU/ml	None detected
B	Frozen	DL3 5×10^6/kg	22.6	99.3	122	No (120)	No growth	≤2EU/ml	None detected

Table S6: Maximum CTCAE grade adverse events observed within 28 days of ATIMP infusion.

Organ Class and AE Name	Maximum AE grade (N=9) 1	2	3	4
**Any Adverse Event**				
Any Adverse Event	.	.	.	9 (100%)
**Blood and lymphatic system disorders**				
Any Blood and lymphatic system disorders	.	1 (11%)	8 (89%)	.
Anemia	.	3 (33%)	6 (67%)	.
Febrile neutropenia	.	.	5 (56%)	.
Lymph node pain	1 (11%)	.	.	.
**Cardiac disorders**				
Any Cardiac disorders	1 (11%)	.	.	.
Sinus tachycardia	1 (11%)	.	.	.
**Eye disorders**				
Any Eye disorders	3 (33%)	.	.	.
Dry eye	1 (11%)	.	.	.
Blurred vision	1 (11%)	.	.	.
Vitreous hemorrhage	1 (11%)	.	.	.
**Gastrointestinal disorders**				
Any Gastrointestinal disorders	4 (44%)	.	3 (33%)	.
Nausea	2 (22%)	.	1 (11%)	.
Vomiting	2 (22%)	.	1 (11%)	.
Abdominal pain	1 (11%)	1 (11%)	.	.
Diarrhea	3 (33%)	1 (11%)	1 (11%)	.
Stomach pain	1 (11%)	.	.	.
Anal pain	1 (11%)	.	.	.
Hemorrhoids	1 (11%)	.	.	.
**General disorders and administration site conditions**				
Any General disorders and administration site conditions	3 (33%)	4 (44%)	.	.
Fatigue	3 (33%)	1 (11%)	.	.
Chills	.	1 (11%)	.	.
Fever	1 (11%)	1 (11%)	.	.
Edema limbs	.	1 (11%)	.	.
Neck edema	1 (11%)	.	.	.
**Immune system disorders**				
Any Immune system disorders	2 (22%)	2 (22%)	.	.
Cytokine release syndrome	2 (22%)	2 (22%)	.	.
Other Immune system disorders: Hypogammaglobulinemia	1 (11%)	.	.	.
**Infections and infestations**				
Any Infections and infestations	1 (11%)	3 (33%)	2 (22%)	.
Urinary tract infection	.	.	1 (11%)	.
Tooth infection	.	.	1 (11%)	.
Other Infections and infestations: Unknown	.	1 (11%)	.	.
Other Infections and infestations: Streptococcus oralis	.	1 (11%)	.	.
Lung infection	.	1 (11%)	.	.
Penile infection	.	1 (11%)	.	.
Other Infections and infestations: Streptococcus gordonii	.	.	1 (11%)	.
Other Infections and infestations: Parainfluenza	1 (11%)	.	.	.
Skin infection	.	1 (11%)	.	.

Organ Class and AE Name	Maximum AE grade (N=9)			
	1	2	3	4
**Injury, poisoning and procedural complications**				
Any Injury, poisoning and procedural complications	1 (11%)	.	.	.
Fall	1 (11%)	.	.	.
**Investigations**				
Any Investigations	.	.	.	9 (100%)
Neutrophil count decreased	.	.	.	9 (100%)
White blood cell decreased	.	.	.	7 (78%)
Lymphocyte count decreased	.	.	.	8 (89%)
Platelet count decreased	.	.	1 (11%)	8 (89%)
Lymphocyte count increased	.	1 (11%)	.	.
Alanine aminotransferase increased	1 (11%)	.	.	.
Other Investigations: Raised Ferritin	1 (11%)	.	.	.
Weight gain	1 (11%)	.	.	.
**Metabolism and nutrition disorders**				
Any Metabolism and nutrition disorders	2 (22%)	2 (22%)	.	.
Hypokalemia	1 (11%)	1 (11%)	.	.
Hypophosphatemia	.	2 (22%)	.	.
Anorexia	1 (11%)	.	.	.
Hypocalcemia	1 (11%)	.	.	.
Hyponatremia	1 (11%)	.	.	.
**Musculoskeletal and connective tissue disorders**				
Any Musculoskeletal and connective tissue disorders	2 (22%)	.	.	.
Neck pain	1 (11%)	.	.	.
Joint range of motion decreased	1 (11%)	.	.	.
**Nervous system disorders**				
Any Nervous system disorders	4 (44%)	1 (11%)	.	.
Lethargy	.	1 (11%)	.	.
Headache	4 (44%)	.	.	.
Paresthesia	1 (11%)	.	.	.
Somnolence	1 (11%)	.	.	.
**Psychiatric disorders**				
Any Psychiatric disorders	.	.	1 (11%)	.
Insomnia	.	.	1 (11%)	.
**Renal and urinary disorders**				
Any Renal and urinary disorders	1 (11%)	.	.	.
Urinary tract pain	1 (11%)	.	.	.
**Respiratory, thoracic and mediastinal disorders**				
Any Respiratory, thoracic and mediastinal disorders	4 (44%)	2 (22%)	1 (11%)	.
Cough	4 (44%)	1 (11%)	.	.
Dyspnea	2 (22%)	1 (11%)	.	.
Pharyngeal mucositis	1 (11%)	.	.	.
Pleural effusion	.	.	1 (11%)	.
Laryngeal inflammation	2 (22%)	.	.	.
Sore throat	2 (22%)	.	.	.
**Skin and subcutaneous tissue disorders**				
Any Skin and subcutaneous tissue disorders	3 (33%)	1 (11%)	.	.
Rash maculo-papular	1 (11%)	1 (11%)	.	.
Purpura	1 (11%)	.	.	.
Erythema multiforme	1 (11%)	.	.	.

Table S7: Tabulated results of 6- and 12-month survival rates (all patients). Key: * NE = Not Evaluable; OS (Overall survival). EFS (Event-free survival). OS event = all-cause mortality. EFS event = relapse or all-cause mortality. Median FUP amongst dead patients: 16.1 months [n=7]. Median FUP amongst patients with no dead reported: 32.7 months [n=2]

	OS	***EFS***
Total	n = 9	n = 9
Number of events	7	7
Median time to event in months (95% CI)	17.2 (5.3 to 31.6)	3.1 (1.8 to 30.4)
6 month rate (95% CI)	78% (36% - 94%)	33% (8%-62%)
12 month rate (95% CI)	67% (28% - 88%)	33% (8%-62%)

SUPPLEMENTAL FIGURES

Figure S1

(a)

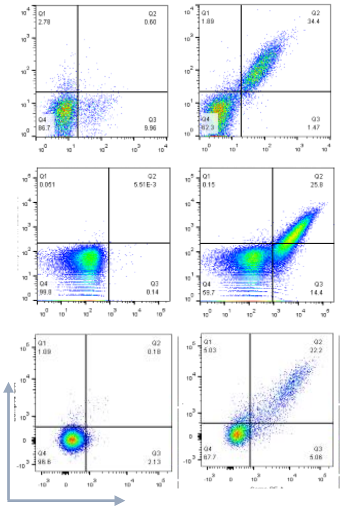

(b)

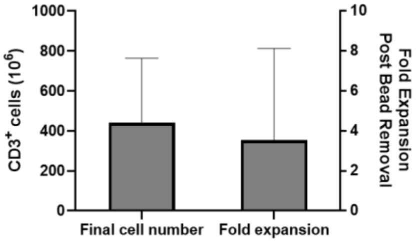

(c)

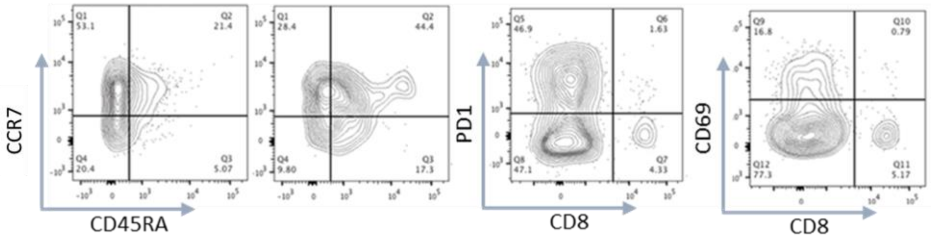

(d)

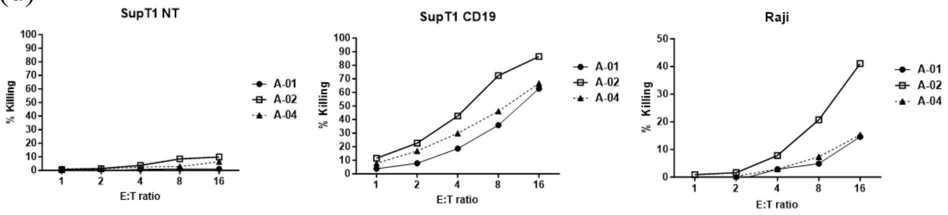

(e)

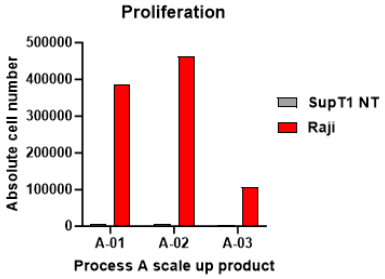

(f)

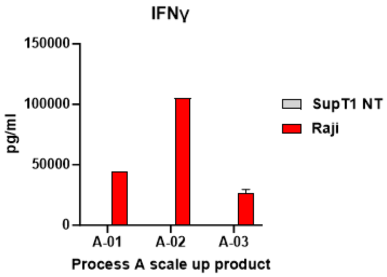

Figure S1: Process A CAR-T product scale-ups using healthy donor PBMCs. Figure S1a: Flow cytometric analysis of scale-up runs. A sample of the cell products compared with NT controls were stained prior to cryopreservation with APC conjugated QBEND/10 and PE-conjugated polyclonal anti-murine-F(ab). The former detects RQR8, while the latter detects the CAR. Cells were analysed by flow-cytometry and fluorescence from QBEND/10 and poly-clonal anti-murine-F(ab) could be independently detected and is shown here as a dot plot with QBEND/10 signal on the y-axis and anti-F(ab) signal on the x-axis. Co-expression of both transgenes is detected. **Figure S1b:** Final cell counts, and fold expansion of the scale-up products. **Figure S1c:** Extended phenotyping of CAR-T cells in an exemplar manufacture process post-thaw using antibodies against CD4, CD8, CCR7, CD45RA, CD69 and PD-1. **Figure S1d:** Chromium release assays were performed against targets: SupT1 NT, SupT1.CD19 and Raji cells at vary effector:target ratios. Post-thaw CAR T-cell products killed SupT1.CD19 and Raji target cells in a dose dependant manner but not SupT1 NT cells. **Figure S1e:** CAR T-cell products proliferate robustly in response to Raji cells following 7-day co-culture. **Figure S1f:** CAR T-cell products produce IFNγ in response to Raji cells in co-culture. Supernatant collected for testing at day 3 of co-culture.

Figure S2

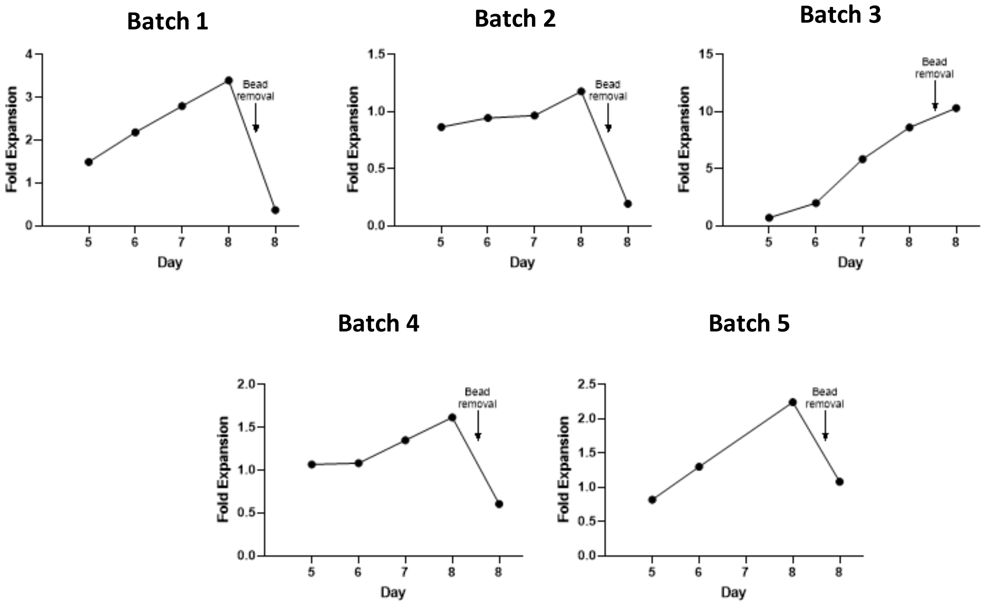

Figure S2: Expansion data for each patient run carried out with Process A. White blood cell (WBC) counts were carried out as in-process controls with the use of a haematological counter (Sysmex). This data reveals the variability of Process A with patient starting material, with a single batch reaching adequate levels of expansion, while 4/5 showed limited cell growth, followed by significant losses during bead removal.

Figure S3

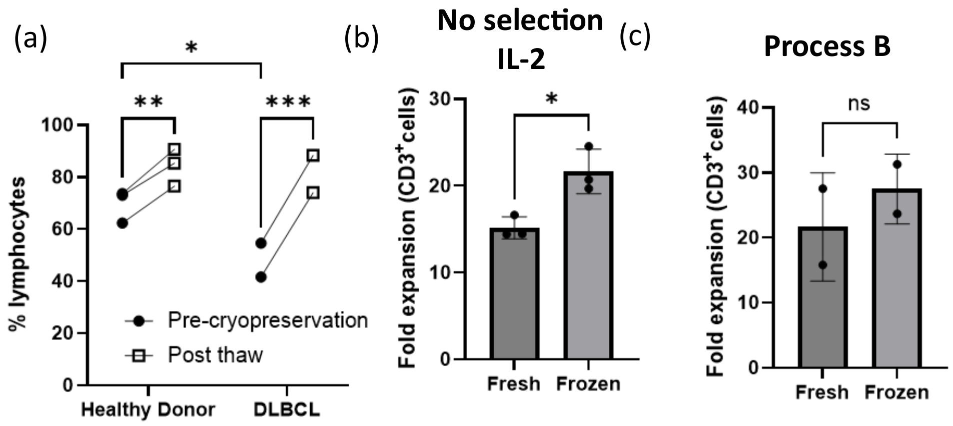

Figure S3: Impact of starting material cryopreservation on manufacturing outcomes. **Figure S3a:** Fold expansion in COBALT batches manufactured using Process B from fresh or frozen patient starting material. Bars represent mean ± SE, Student's t-test, p <0.05. **Figure S3b:** Fold expansion in 4g7 CAR T batches manufactured on the CliniMACS Prodigy using from healthy donors Process B, but with no T cell enrichment step and 100 IU/mL IL-2, instead of 10 ng/mL IL-7/15. Bars represent mean ± SE, Student's t-test, p <0.05. **Figure S3c:** Lymphocyte % obtained from haematological counter (Sysmex) before starting material cryopreservation and or after thaw. LBCL or healthy donor leukapheresis was cryopreserved following standard local procedures. They were thawed in TexMACS medium with 10% human AB serum and rested overnight at 37oC, 5% CO2 without cytokines, before manufacture initiation. Analysis carried out by two-way RM ANOVA

Figure S4

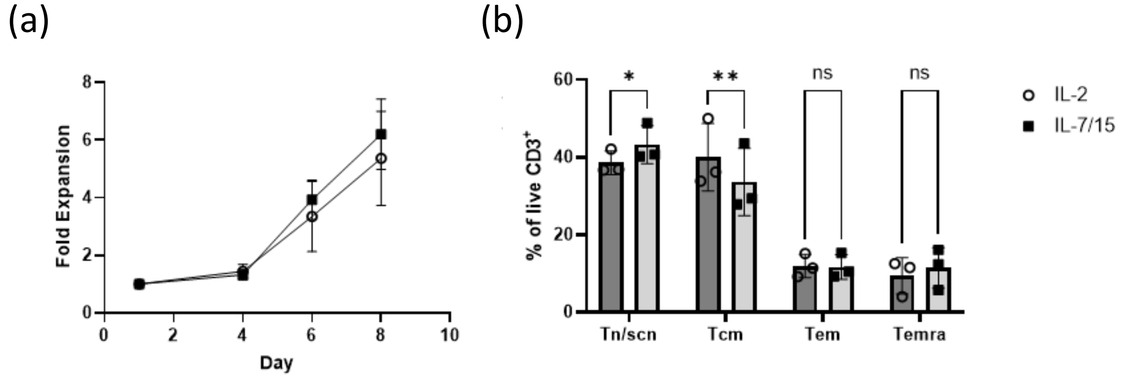

Figure S4: Comparison between IL-2 and a combination of IL-7and IL-15 on T cell expansion and phenotype. Healthy donor cells were obtained from peripheral blood of volunteers. PBMCs were enriched with a Ficoll density gradient and T cells were labelled with the CliniMACS CD4 and CD8 reagents (Miltenyi, 200-070-132 and 200-070-115). They were selected using LS columns (Miltenyi, 130-042-401) with a QuadroMACS™ magnet. Cells were seeded at 1 x 106 CD3+/mL in TexMACS 3% human AB serum and activated using 10 µL/mL human T cell TransAct (Miltenyi, 130-111-160), and 100 IU/mL IL-2 (Miltenyi, 130-097-745) or 10 ng/mL IL-7 (Miltenyi, 130-095-362) and 10 ng/mL IL-15 (Miltenyi, 130-095-764). A culture wash was carried out on day 4, and cells kept at a density of 2 x 106/mL in TexMACS 3% human AB serum and the relevant cytokines, with media exchanges performed every 2 days. **Figure S4a:** T cell expansion in cultures using 100 IU/mL IL-2, or 10 ng/mL IL-7/IL-15. No differences were observed in T cell growth. Plots show represent mean ± SE. **Figure S4b:** Memory phenotype evaluated at the end of culture (day 8). The use of IL-7/15 resulted in a discrete increase in the number of Tn/scm with consequent decrease in Tcm subsets. These results indicate that the combination of IL-7 and IL-15 can maintain the same level of T cell expansion observed with IL-2 while retaining a less differentiated phenotype. Bars represent mean ± SE. Analysis carried out by two-way RM ANOVA with Šídák's multiple comparisons test.

Figure S5

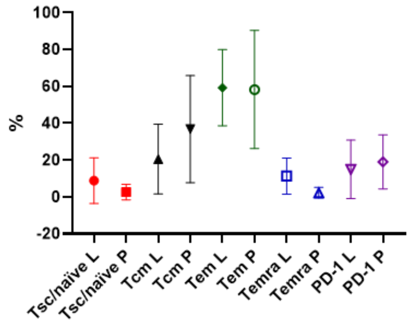

Figure S5: COBALT patient 4G7CAR-T product manufactures. Evolution of memory and exhaustion markers by flow cytometry from leukapheresis starting material (L) to final 4G7CAR-T product (P) on Process-B. T cell subsets defined as previously. Graphs show mean ± SD.

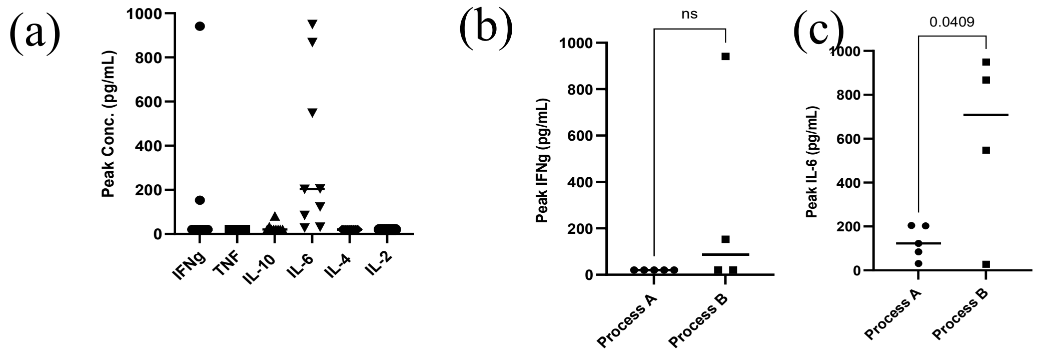

Figure S6: COBALT study cytokine analysis. **Figure S6a:** Summary of peak cytokines for all patients. **Figure S6b:** Comparison of peak IL-6, peak IFNg between Process-A and Process-B.

REFERENCES

1. Ghorashian S, Kramer AM, Onuoha S, Wright G, Bartram J, Richardson R, Albon SJ, Casanovas-Company J, Castro F, *et al.* Enhanced CAR T cell expansion and prolonged persistence in pediatric patients with ALL treated with a low-affinity CD19 CAR. *Nat. Med.* **25**, 1408–1414 (2019).
2. Dull T, Zufferey R, Kelly M, Mandel RJ, Nguyen M, Trono D, Naldini L. A Third-Generation Lentivirus Vector with a Conditional Packaging System. *J. Virol.* **72**, 8463–8471 (1998).
3. Lee DW, Santomasso BD, Locke FL, Ghobadi A, Turtle CJ, Brudno JN, Maus MV, Park JH, Mead E, Pavletic S, *et al.* ASTCT Consensus Grading for Cytokine Release Syndrome and Neurologic Toxicity Associated with Immune Effector Cells. *Biology of Blood and Marrow Transplantation* **25**, 625–638 (2019).
4. MoCA Montreal - Cognitive Assessment. *MoCA Montreal - Cognitive Assessment* https://www.mocatest.org/.
