# Supplementary material for: A comparison of two manufacturing methods in the phase I COBALT study of CD19CAR T for LBCL
Source: Mol Ther Adv. 2026 Feb 13;34(1):201695. doi: 10.1016/j.omta.2026.201695 (PMC13148904; doi:10.1016/j.omta.2026.201695)
Supplement: Document S2. Article plus supplemental information [file mmc2.pdf]

Molecular Therapy
Advances
Original Article

A comparison of two manufacturing methods in the phase I COBALT study of CD19CAR T for LBCL

Claire Roddie,[1,2] Juliana Dias,[1,3] Gordon Weng-Kit Cheung,[1] Maeve A. O'Reilly,[2] Mahnaz Abbasian,[1] Amaia Cadinanos-Garai,[1] Ketki Vispute,[1] Leticia Bosshard-Carter,[1] Marina Mitsikakou,[1] Vedika Mehra,[1] Harriet Roddy,[1] John A. Hartley,[1,6] Nasir G. Mahmoud,[1,6] Leah Ensell,[1,6] Yashma Patel,[1,6] Maria A.V. Marzolini,[2] Farzin Farzaneh,[4] Nourredine Himoudi,[7] Farhatullah Syed,[7] Bilyana Popova,[5] Andre Lopes,[5] Alexander Day,[5] Mark W. Lowdell,[1,3] and Karl S. Peggs[1,2]

[1]Cancer Institute, University College London, London, UK; [2]Department of Haematology, University College London Hospitals, London, UK; [3]Centre for Cell/Gene/Tissue Therapeutics (CCGTT) Royal Free Hospital London, London, UK; [4]Gene Vector Laboratory, Kings College London, London, UK; [5]CRUK UCL Cancer Trials Centre, London, UK; [6]UCL Experimental Cancer Medicine Centre Good Clinical Laboratory Practice Facility, London, UK; [7]UCL Institute of Child Health (ICH), London, UK

As demand for CAR T products increases, finding solutions to manufacturing bottlenecks becomes critical. While most current FDA-approved CAR T products are manufactured using traditional bag-based manufacturing methods, semi-automated manufacturing platforms can simplify and expedite autologous CAR T product delivery to patients. We performed the phase I COBALT study (NCT02431988) of 2nd-generation CD19 CAR T cells for relapsed/refractory (r/r) large B cell lymphoma (LBCL). Here, we compared a manual, bag-based, IL2-supplemented manufacture process (process-A) with a CD4/8-preselected, semi-automated, interleukin (IL)-7/IL-15-supplemented Miltenyi CliniMACS Prodigy-based manufacturing process (process-B), with a focus on the drug product and manufacturing feasibility and logistics. GMP scale-up runs using leucapheresis products from people with LBCL showed that process-B delivered the target CAR T dose more consistently than process-A, with lower viral vector usage, less grade A clean room time, and less hands-on staff time required per product. On study, ten patient-specific products were manufactured (5 with process-A; 5 with process-B). 6 of 10 products reached the target dose (2 of 5, process-A; 4 of 5, process-B), and 9 of 10 patients were infused. Higher early CAR T expansion was observed in patients treated with process-B products. In this analysis within the COBALT study, process-B compares favorably with process-A in reproducibly reaching the target CAR T dose in people with r/r LBCL, and appears to be associated with better CAR T expansion *in vivo*.

INTRODUCTION

Clinical trials and real-world experience of CD19-directed chimeric antigen receptor T cells (CAR T) have demonstrated sustained responses in adults with relapsed/refractory (r/r) large B cell lymphoma (LBCL),[1–3] but as demand for CAR T products and clinical trials increases, systems enabling large-scale CAR T manufacturing capability become increasingly important.[4] CAR T manufacturing is a multi-step process of cell isolation, activation, transduction, expansion, and cryopreservation. Multiple factors, including cell composition of starting material collected from patients,[5,6] duration of T cell culture,[7] and cytokine supplementation,[8] can impact product potency, independent of CAR design.[9] Barriers to scaling up include complex manufacturing protocols, high costs, and a shortage of good manufacturing practice (GMP)-compliant clean rooms and highly skilled staff.[10,11]

Here, we designed and developed a 2nd-generation CD19-directed CAR[12] from the 4G7 hybridoma,[13] henceforth referred to as 4G7CAR T. Using healthy donor leukapheresis, we validated a manual, bag-based manufacturing protocol incorporating CTS Dynabead CD3/CD28 T cell activation, interleukin-2 (IL-2), and WAVE bioreactor-based T cell expansion, and commenced the academic phase I COBALT (NCT02431988) trial of 4G7CAR T in people with r/r LBCL.

In line with evolution in manufacturing practice at our center during the first half of the study, we optimized our manufacturing workflow and initiated a head-to-head comparison of our original bag-based, manual, multi-operator, IL-2-supplemented manufacturing process (process-A) with a semi-automated, closed, IL-7/IL-15-supplemented[14,15] manufacturing process on the Miltenyi CliniMACS Prodigy (process-B) using leukapheresis from patients with LBCL. Process-B additionally incorporated immunomagnetic CD4/CD8 selection prior to activation and viral transduction. The results of the

Received 13 February 2025; accepted 9 February 2026;
https://doi.org/10.1016/j.omta.2026.201695.

Correspondence: Claire Roddie, Cancer Institute, University College London, London, UK.
E-mail: c.roddie@ucl.ac.uk

© 2026 The Author(s). Published by Elsevier Inc. on behalf of The American Society of Gene and Cell Therapy. This is an open access article under the CC BY license (http://creativecommons.org/licenses/by/4.0/).

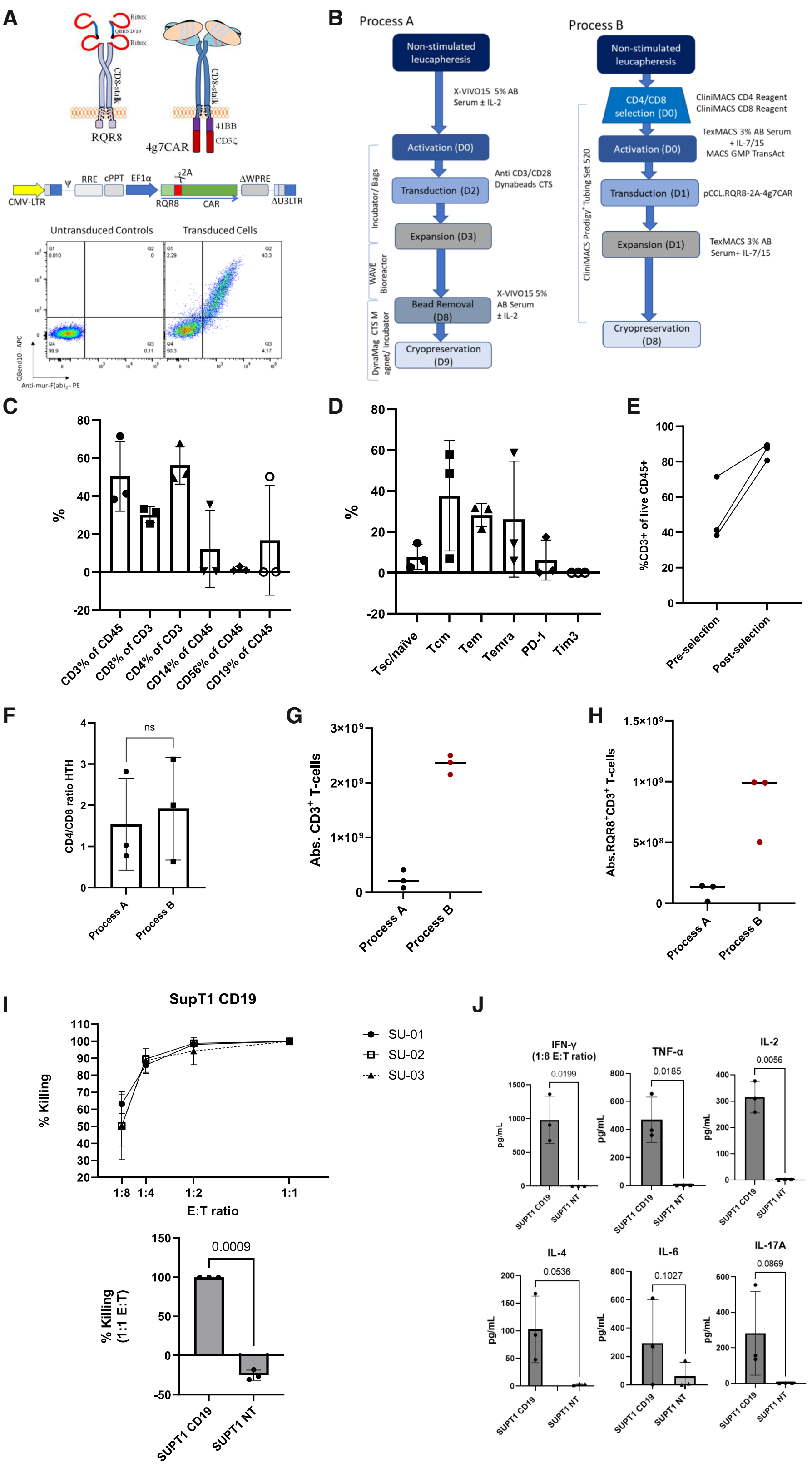

(legend on next page)

head-to-head analysis prompted us to switch from process-A to process-B for the second half of the study.

Here, we assess how the two different manufacturing processes perform in the COBALT study in relation to CAR T product yield, viral vector consumption, and GMP cleanroom and staff requirements per process. We additionally review outcomes from the COBALT study, including 4G7CAR T engraftment, alongside patient safety and preliminary response outcomes.

RESULTS

4G7 CD19 binder/4G7CAR T preclinical development

4G7CAR T preclinical development is as described previously.[12] 4G7CAR and the RQR8 sort-suicide protein[16] are illustrated in cartoon format, alongside the pCCL.EF1a.RQR8-2A-4G7CAR vector in linear plasmid map format. Equimolar expression of 4G7CAR and RQR8 is demonstrated by flow cytometry in transduced healthy donor peripheral blood mononuclear cells (PBMCs) (Figure 1A).

Manufacture validation and scale-up runs for process-A and process-B

Manufacturing process-A (Figure 1B) was validated using leukapheresis material from 3 healthy donors. Scale-up products met the proposed COBALT product release criteria (Table S3), including transduction efficiency (Figure S1A), with products enriched in Tn/scm and Tcm subsets (Figure S1C). All scale-up products demonstrated CD19-specific cytotoxicity (Figure S1D), proliferation (Figure S1E), and IFN-γ secretion (Figure S1F).

Manufacturing process-B (also Figure 1B) scale-up runs were conducted using cryopreserved surplus LBCL leukapheresis material from 3 COBALT patients previously manufactured on process-A. Leukapheresis immune cell composition analysis performed on cryopreserved samples showed that mean CD3+ T cell content was 50.4% (Figure 1C), comprising mostly Tcm and Tem populations, without a prominent signature of exhaustion (Figure 1D).

For process-A, a total of 500–1000 $\times 10^6$ PBMCs were activated without a T cell enrichment step. Between 250–750 $\times 10^6$ cells were then transduced 48 h post-activation, and 2 days later were transferred into a WAVE bioreactor for expansion (Figure 1B).

In contrast, process-B involved activation and transduction of a fixed number (80–100 $\times$ 10^6) of selected T cells prior to cultivation and expansion on the CliniMACS Prodigy (Figure 1B). Figure 1E shows successful T cell enrichment post-CD4/8 immunomagnetic selection, increasing the mean percentage of CD3+ cells from 50.4% pre-selection to 85.9% post-selection.

At the end of manufacturing, while the CD4/CD8 ratio was similar between process-A and process-B (Figure 1F), the absolute $CD3^+$ (Figure 1G) and 4G7CAR+ (Figure 1H) T cell numbers obtained from process-B in all 3 scale-up runs exceeded the target dose requirements for all 3 dose levels on COBALT (dose level [DL]1, 2 $\times$ 10^5/kg; DL2, 1 $\times$ 10^6/kg; DL3, 5 $\times$ 10^6/kg), whereas 2 of 3 process-A runs failed to meet the lower doses (DL1/DL2) despite using the same cellular starting material. This comparative data is presented head-to-head in Table 1. Target-specific potency analysis for process-B scale-up products is shown in Figures 1I and 1J.

From a manufacturing logistics perspective, GMP grade A/B clean room usage for process-B was lower than for process-A, estimated

Figure 1. Process-A vs. process-B scale-up runs using COBALT patient leukapheresis

(A) A schematic of RQR8 and 4G7CAR transgene products alongside a linear plasmid map of the pCCL.EF1a.RQR8-2A-4G7CAR vector. RQR8 is a compact sort-suicide gene comprising two copies of a rituximab-binding peptide (red), flanking a fragment of CD34, which binds QBEND/10, as described previously.[16] 4G7CAR consists of the 4G7 scFv connected to the CD8 stalk and TM domain, as described previously.[12] Donor T cells were transduced with pCCL.RQR8-2A-4G7CAR lentiviral vector and stained for RQR8 (anti-hCD34 QBEND/10 monoclonal antibody) and 4G7CAR (polyclonal anti-murine F(ab)). Non-transduced cells are shown on the left and transduced cells on the right.

(B) Schematic of process-A (bag-based/Dynabead/IL-2) vs. process-B (Prodigy-based/TransAct/IL7-IL15).

(C) Patient with LBCL leukapheresis starting material for head-to-head runs for validation of process B: immune cell composition by flow cytometry. Bars represent mean ± SD.

(D) Patient with LBCL leukapheresis starting material for head-to-head runs for validation of process B: T cell memory/exhaustion phenotypes assessed by flow cytometry (Tn/scm, $CCR7^+CD45RA^+$; Tcm, $CCR7^+CD45RA^-$; Tem, $CCR7^-CD45RA^-$; Temra, $CCR7^-CD45RA^+$). Bars represent mean ± SD.

(E) T cell enrichment for process-B is successfully achieved through CD4/8 immunomagnetic selection (mean %CD3+ pre-selection = 50.4%; mean %CD3+ post-selection = 85.9%).

(F) CD4/CD8 ratio at the end of process-A and process-B. Bars represent mean ± SD. Analysis was carried out using a paired Student's *t* test ($p = 0.7396$).

(G) Absolute $CD3^+$ numbers obtained at end-of-manufacture from scale-up runs using patient with LBCL leukapheresis on process-A vs. process-B. Graphs show median values.

(H) Absolute 4G7CAR T numbers obtained at end-of-manufacture on head-to-head runs using patient with LBCL leukapheresis on process-A vs. process-B (measured by marker gene expression, $RQR8^+$). Graphs show median values.

(I) 24-h FACS-based killing of GFP-SupT1 cells either CD19- (SupT1 NT) or stably expressing CD19 (SupT1 CD19) at different effector-to-target (E:T) ratios for each of the process-B scale-ups (top). % killing was calculated by normalizing the number of GFP^+ targets remaining in the co-culture culture with CAR T products to the number of GFP^+ targets in the co-culture with the corresponding untransduced controls at the same E:T ratio. The bottom panel shows a summary potency analysis for a 1:1 E:T ratio against $CD19^+$ and $CD19^-$ targets (mean ± SD). Analysis was carried out using a paired Student's *t* test ($p = 0.009$).

(J) Production of anti-tumour effector (IFN-γ, TNF-α), stimulatory (IL-2), regulatory (IL-4), and inflammatory (IL-6, IL-17A) cytokines by 4G7CAR T cells generated with process-B, in the presence of CD19- (SupT1 NT) or CD19- (SupT1 CD19) targets. Plots show quantification of each cytokine in the co-culture supernatants after 24 h at a 1:1 E:T ratio, using the LEGENDPlex Human Essential Immune Response Panel. Levels of IFN-γ at a 1:1 E:T ratio exceeded the assay detection limits (>10,000 pg/mL); results obtained at a 1:8 E:T ratio are displayed. Bars show mean ± SD, and analysis was carried out using paired Student's *t* test (*p* values shown in the graphs).

Table 1. Process-A vs. process-B manufacture validation results using COBALT patient with LBCL leukapheresis from 3 patients treated with process-A products, in whom process-B was conducted using surplus leukapheresis material

	Process	Target Dose Level (DL)	CAR % of CD3	CD3 viability %	Total CAR T cell yield ($\times 10^6$)	Target Dose Met (Y/N)	Sterility (no growth 10d)	Endotoxin	Mycoplasma
Leuka X	A	DL1 2 x 10^5/kg	18.1	94.5	13.6	Y	no growth	≤2EU/ml	none detected
	B	DL1 2 x 10^5/kg	21.2	99.8	502	Y	no growth	≤2EU/ml	none detected
Leuka Y	A	DL2 1 x 10^6/kg	13.7	95.8	56.2	N	no growth	≤2EU/ml	none detected
	B	DL2 1 x 10^6/kg	39.6	99.7	991	Y	no growth	≤2EU/ml	none detected
Leuka Z	A	DL2 1 x 10^6/kg	15.3	99	64.9	N	no growth	≤2EU/ml	none detected
	B	DL2 1 x 10^6/kg	46.2	99.9	993	Y	no growth	≤2EU/ml	none detected

at 8 vs. 29 h. Further, total operator hours for process-B were also lower (18 vs. 35 h), and the required clean room footprint was smaller, i.e., incubators, WAVE bioreactors, and CTS DynaMags were all replaced by a single CliniMACS Prodigy device. Of note, viral vector volume requirements were up to 7.5-fold lower for process-B, due to the lower starting cell numbers stipulated for manufacturing on day 0 (process-B, 100 $\times$ 10^6 T cells; process-A, 250–750 $\times$ 10^6 total cells). This is elaborated further in Table 2.

Patient and disease characteristics

10 patients with LBCL were registered, enrolled, and underwent leukapheresis in the COBALT study, and 9 of 10 were infused with 4G7CAR T. 1 patient did not proceed to infusion due to pre-LD progression and infection.

The median age of patients in COBALT was 50 years (range, 44–62), and most patients had high-risk disease (56% primary refractory; 78% baseline International Prognostic Index (IPI) 3–5; 100% baseline Lactate Dehydrogenase (LDH) $\geq$ upper limit of normal (ULN)[17,18]). Patients had received a median of 4 prior lines of therapy (range, 3–6), including one patient who had failed prior CD19CAR T (Tisagenlecleucel) but retained CD19 expression by immunohistochemistry. Patient demographics and bridging data are listed in Tables 3 and S4.

4G7CAR T cell patient manufacturing outcomes

10 patient products were manufactured: 5 on process-A and 5 on process-B. The median CD3% in the leukapheresis was 62% (range, 16–72), with a median CD8, CD4, and monocyte fraction of 29% (range, 17–50), 54% (46–77), and 14% (range, 0.37–40), respectively. Separating patient leukapheresis data into process-A and process-B (black vs. red points, respectively), we demonstrate no overt differences in composition or memory/exhaustion phenotypes between processes (Figures 2A and 2B). While end-of- manufacturing transduction efficiency was not significantly different between processes (Figure 2C), mean absolute CD3+ T cell numbers (Figure 2D) and absolute 4G7CAR T yield (Figure 2E) were higher in process-B than in process-A (507 $\times$ 10^6 vs. 123 $\times$ 10^6 total 4G7CAR T cells).

All products were released by the qualified person (QP), but only 2 (of 5) process-A products met the target dose, despite including patients recruited to the lowest DL cohort (DL1), whereas 4 (of 5) process-B products met the target dose, despite including patients recruited to the highest DL (DL3). This information is presented in detail in Table S5.

Exploration of 4G7CAR T cell patient manufacturing outcomes on process-A

While process-A scale-up runs using healthy donor leukapheresis demonstrated feasibility, reaching the proposed specification for 4G7CAR T products to be released in the study (Figure S1) manufacturing outcomes with leukapheresis from heavily pretreated adult patients with LBCL were very different. Figure S2 shows the expansion profile for each COBALT 4G7CAR T batch manufactured using process-A. Despite IL-2 supplementation, we observed limited cell expansion (median, 2.2-fold; range, 1.2–8.6) during the 4 day WAVE bioreactor incubation. This was accompanied by low cell recovery after magnetic removal of CTS Dynabeads (median recovery, 37%; range, 10.8%–119.6%), further contributing to the lower cell numbers obtained with process-A.

While it is possible that patient features or prior treatments impacted manufacturing feasibility, Table 2 shows that demographics, including prior therapeutic lines (Table S4), were similar between process-A and process-B patients (Table 2). The only process-B product failing to reach the COBALT target dose (DL3) was derived from a patient who had been exposed to 3 cycles of bendamustine-containing chemotherapy prior to leukapheresis. It is recognized that recent exposure to bendamustine is associated with impaired T cell fitness and worse CAR T clinical outcomes.[19]

It is also possible that some of the differences in 4G7CAR T yields from process-B vs. process-A relate to differences in the handling

Table 2. Process-A vs. process-B comparison of staff time, clean room time, reagents, consumables, and equipment

		Reagent Prep.	CD4/CD8 selection	Culture setup/T cell activation	Transduction	Transfer to WAVE bioreactor	Expansion	Bead removal	Freeze
**Process A**	Grade A<br>Hands-on<br>Staff no.	5 h<br>6 h<br>2	N/A<br>N/A<br>N/A	4 h<br>5 h<br>3	3 h<br>3 h<br>2	4 h<br>6 h<br>3	3 h<br>3 h<br>2	5 h<br>6 h<br>3	5 h<br>6 h<br>3
	**Total Hours/process for Process A:**<br>Grade A: **29 h**<br>Hands-on: **35 h**								
	additional reagents	N/A	N/A	Dynabeads<br>cultivation bags	cultivation bags<br>retronectin<br>consumables for cell wash/feed<br>higher volume of vector required (2.5–7.5 fold)	WAVE bags<br>2L X-VIVO15<br>MACS GMP recombinant human IL-2	2L X-VIVO15<br>MACS GMP recombinant human IL-2	CliniMACS PBS/EDTA+HAS<br>X-VIVO15<br>MACS GMP recombinant human IL-2	consumables for cell washing and sampling
	equipment required	plasma therm	N/A	incubator	incubator	incubator<br>WAVE Bioreactor	WAVE Bioreactor	DynaMag CST magnet<br>WAVE Bioreactor	CRF
		reagent preparation	CD4/CD8 selection	culture setup/T cell activation	transduction	transfer to WAVE bioreactor	expansion	beads removal	freeze
**Process B**	grade A<br>hands-on<br>staff	2 h<br>4 h<br>2	2 h<br>5 h<br>2	N/A<br>1 h<br>2	1 h<br>1 h<br>2	N/A<br>N/A<br>N/A	N/A<br>2 h<br>2	N/A<br>N/A<br>N/A	3 h<br>5 h<br>3
	**Total Hours/process for Process B:**<br>Grade A: **8 h**<br>Hands-on: **18 h**								
	Additional reagents	N/A	CliniMACS<br>CD4 Reagent<br>CliniMACS<br>CD8 reagent<br>TS 520 tubing set	MACS GMP<br>T cell TransAct	N/A	N/A	2.5L TexMACS 3% AB serum<br>MACS GMP recombinant human IL-15<br>MACS GMP recombinant human IL-7	N/A	N/A (Cell wash and harvest performed by the Prodigy)
	Equipment required	sterile tubing welder	CliniMACS Prodigy	CliniMACS Prodigy<br>sterile tubing welder	CliniMACS Prodigy<br>sterile tubing welder	N/A	CliniMACS Prodigy<br>sterile tubing welder	N/A	CRF

CRF, controlled rate freezer; HAS, human albumin solution; PBS, phosphate-buffered saline.

Table 3. Infused patient demographics on the COBALT study

Baseline Characteristics	*Overall (N = 9 (%)*	*Process A (N = 5)*	*Process B (N = 4)*
Sex			
Female	2 (22%)	1 (20%)	1 (25%)
Male	7 (78%)	4 (80%)	3 (75%)
Median age in years (range)	50 (43–62)	51 (49–59)	49 (43–62)
Disease characteristics, n (%)*			
Transformed Follicular Lymphoma (tFL)	5 (56%)	2 (40%)	3 (75%)
De novo DLBCL	4 (44%)	3 (60%)	1 (25%)
DHL/THL	0 (0%)	0 (0%)	0 (0%)
Primary refractory disease	5 (56%)	4 (80%)	1 (25%)
Extranodal sites	7 (78%)	3 (60%)	4 (100%)
Baseline IPI			
1–2	2 (22%)	2 (40%)	0 (0%)
3–5	7 (78%)	3 (60%)	4 (100%)
Prior Lines of treatment, n			
Median (range)	4 (3–6)	3	4.5
Prior Autologous HSCT, n (%)	1 (11%)	0 (0%)	1 (25%)
Baseline LDH, IU/L			
Median	292	289	415
Range	271–624	271–481	283–624
CNS involvement			
Yes	0 (0%)	0 (0%)	0 (0%)
No	9 (100%)	5 (100%)	4 (100%)
Disease burden prior to lymphodepletion			
Stage I-II	1 (11%)	1 (20%)	0 (0%)
Stage III-IV	8 (89%)	4 (80%)	4 (100%)
Prior CD19CAR T therapy			
Yes (Tisagenlecleucel)	1 (11%)	0 (0%)	1 (25%)
No	8 (89%)	5 (100%)	3 (75%)
Karnofsky performance status			
100	0 (0%)	0 (0%)	0 (0%)
90	1(11%)	0 (0%)	1 (25%)
80	5(56%)	3 (60%)	2 (50%)
70	3(33%)	2 (40%)	1 (25%)
60	0(%)	0 (0%)	0 (0%)

DHL, double hit lymphoma; THL, triple hit lymphoma; IPI, international prognostic index; HSCT, haematopoeitic stem cell transplant; CNS, central nervous system.

of leukapheresis starting material and the cytokines used between processes. For instance, process-A used entirely fresh leukapheresis, but validation runs for process-B were carried out using cryopreserved surplus starting material from the process-A runs. Furthermore, 2 of the 5 clinical production runs on process-B were conducted using cryopreserved leukapheresis. Figure S3A shows significant enrichment for lymphocytes by Sysmex post-cryopreservation in both heathy donor and LBCL starting material. When healthy donor T cells are expanded in the CliniMACS Prodigy without a T cell enrichment step and in the presence of IL-2, superior T cell enrichment and expansion are observed using cryopreserved vs. fresh starting material (Figure S3B). In contrast, we saw no differences in T cell expansion between cryopreserved vs. fresh leukapheresis from patients with LBCL on process-B, which incorporates a T cell enrichment step (Figure S3C). Use of cryopreserved starting material was thus introduced to manufacturing workflows at the time process-B was implemented to permit scheduling flexibility and enhance manufacturing capacity.

Testing IL-2 vs. IL-7/IL-15 in small-scale 8-day culture experiments, we showed that IL-7/IL-15 is associated with a modest increase in Tn/Tscm populations, with a parallel reduction in Tcm populations, but without an appreciable impact on T cell expansion (Figure S4). While these data are not definitive, they suggest that switching cytokines is not wholly responsible for the differences observed in T cell yield between process-A and process-B.

4G7CAR expansion and persistence

A total of 9 patients were infused with 4G7CAR T in the study: 5 patients with products from process-A at DL1 (n = 3) and DL2 (n = 2) and 4 patients with products from process-B at DL2 (n = 3) and DL3 (n = 1). Figure 3B shows durable high-level 4G7CAR T persistence by flow cytometry in a responding patient treated with a process-B product. Flow-marking data for all patients are illustrated in Figure 3C. Peak expansion by flow was higher in patients receiving process-B vs. process-A products (Figure 3D), with the caveat that 3 of 5 process-A patients were treated at DL1. Persistence was demonstrated by qPCR at last follow-up in 8 of 9 patients at a median of 2 months (range, 2–24 months) (Figure 3E), and there was a trend toward higher peak expansion by qPCR for process-B vs. process-A products (Figure 3F).

Toxicity and biological correlates

In this dose-escalation study, 3 of 9 patients were infused at DL1 (2×10^5 4G7CAR T/kg), and no dose-limiting toxicities (DLTs) were observed. 4G7CAR T products for the first 2 patients recruited into DL2 (1×10^6 4G7CAR T/kg) were manufactured by process-A but failed to meet the target dose, such that DLTs could not be assessed. For this reason, a further 3 patients were recruited and treated at DL2, with products manufactured on process-B, all of which met the target dose, and no DLTs were reported. Only one patient was infused at DL3 (5×10^6 4G7CAR T/kg), and again, no DLTs were observed.

Across all dose levels, 4 of 9 infused patients (44%) experienced cytokine release syndrome (CRS) at a median of 3 days post-4G7CAR T (range, 1–7), with 2 grade 2 events.[20] No $\geq$ grade 3 neurotoxicity was reported in the study. Cytokine analysis was conducted for all patients from day −6 until day 28 post-infusion (Figures 3G and

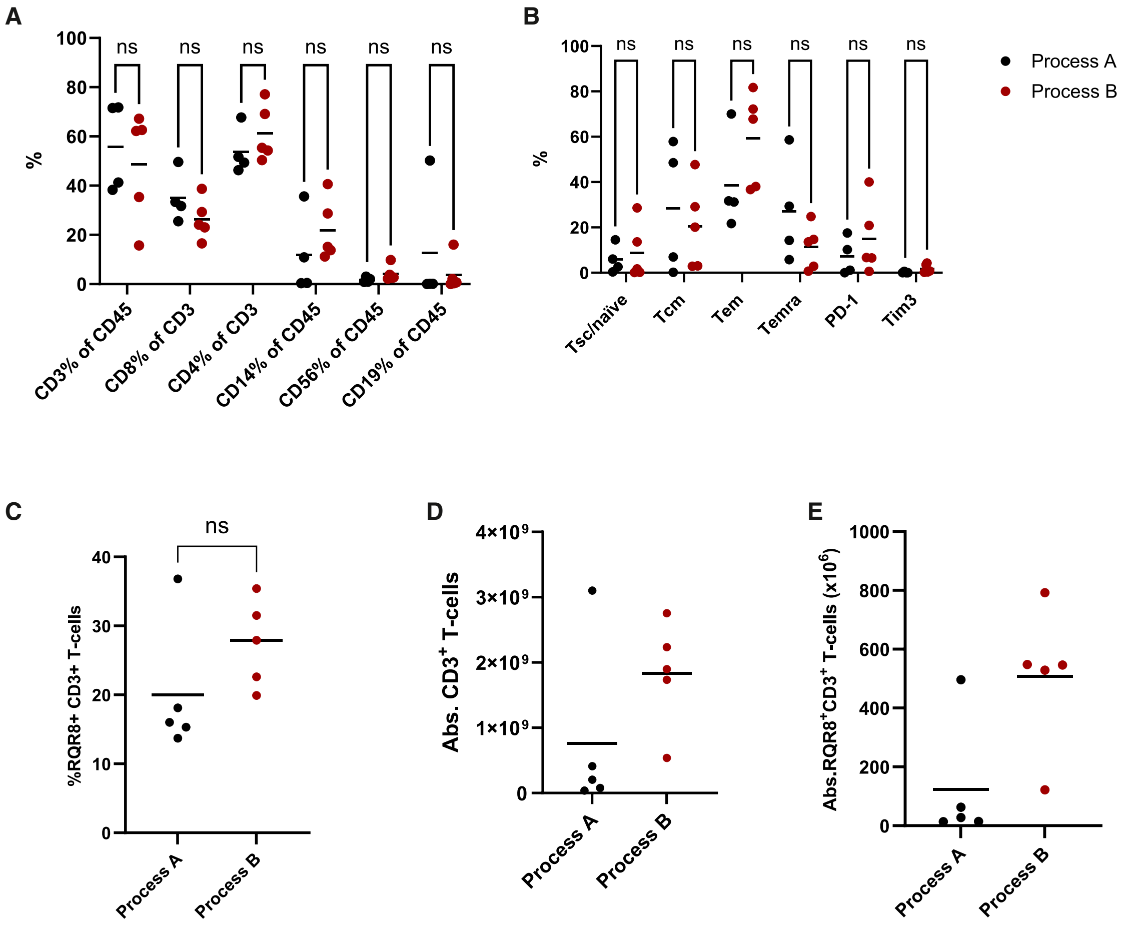

Figure 2. COBALT patient 4G7CAR T product manufactures

(A) Leukapheresis starting material immune cell composition by flow cytometry is shown for all patients and separated into process-A (black points) and process-B (red points). The mean is indicated in the graphs. Analysis was carried out using two-way ANOVA and Šídák's multiple comparisons test.

(B) Leukapheresis starting material memory/exhaustion marker composition by flow cytometry is shown for all patients and separated into process-A (black points) and process-B (red points). T cell subset characterization is as follows: Tn/scm, $CCR7^{+}CD45RA^{+}$; Tcm, $CCR7^{+}CD45RA^{-}$; Tem, $CCR7^{-}CD45RA^{-}$; Temra, $CCR7^{-}CD45RA^{+}$). Graphs indicate the mean. Analysis was carried out using two-way ANOVA and Šídák's multiple comparisons test.

(C) Transduction efficiency obtained with process-A vs. process-B, determined by $\%RQR8^{+}$ cells by flow cytometry. Comparison was carried out by Student's *t* test ($p = 0.182$).

(D) Absolute $CD3^{+}$ numbers obtained at end-of-manufacture from COBALT patient runs on process-A (black lines) vs. process-B (red lines). Graphs indicate the mean.

(E) Absolute 4G7 CAR T cells ($RQR8^{+}$) numbers obtained at end-of-manufacture from COBALT patient runs on process-A (black lines) vs. process-B (red lines). Graphs indicate the mean.

3H). Peak cytokines for all patients are illustrated in Figure S6A, and peak IL-6 was higher with process-B (Figure S6B).

All infused patients experienced grade 3/4 neutropenia and thrombocytopenia during the first 28 days post-4G7CAR T. Only 2 patients had ≥ grade 3 neutropenia beyond day 28. Hypogammaglobulinemia was reported in 8 of 9 infused patients (pre-existing in 3 patients), and 4 of 9 received intravenous immunoglobulin (IVIG). In terms of late infections, the patient died of COVID-19 pneumonia and multi-organ failure at month 30 post-infusion. Two further patients died of infection in the context of subsequent lines of chemotherapy for progressive disease (PD) post-CAR T. Toxicity is summarized in Table 4, and all adverse events in the study are listed in Table S7.

Response rates and survival

Individual patient responses are illustrated in a swimmer's plot in Figures 4A. A tabulated summary of responses is outlined in Tables 4 and S7.

The overall response rate (ORR) at month 1 was 7/9 (78%). Complete metabolic response (CMR) was observed in 3/9 (33%) patients and was ongoing beyond 24 months in all: 2 of 3 without further therapy and 1 of 3 following allogeneic stem cell transplant (allo-SCT) at month 2. Partial response (PR) was reported in 4/9 (44%) patients at month 1, but all progressed within 2–3 months of infusion: 2 with CD19− and 2 with CD19+ disease. Stable disease (SD) or PD was reported in 2/9 (22%) patients.

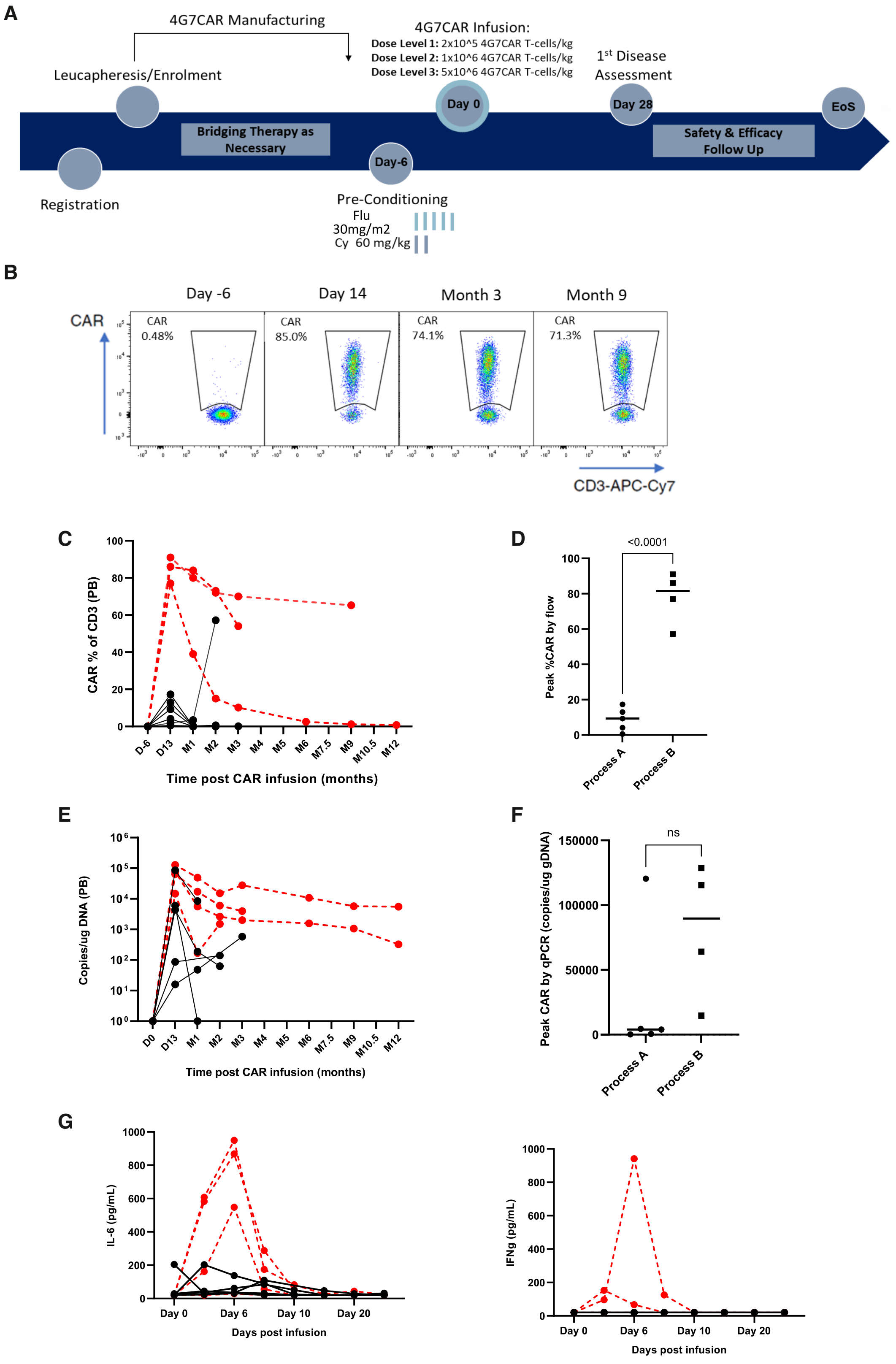

(legend on next page)

The 6- and 12-month overall survival (OS) was 78% (95% confidence interval [CI], 36%–94%) and 67% (95% CI, 28%–88%), and the 6- and 12-month progression-free survival (PFS) was 33% (8%–62%) and 33% (8%–62%) (Figure 4B).

7/9 patients died in the study: 1/7 died in remission post-CAR T with COVID-19 pneumonia and multi-organ failure; 1/7 died from complications of allo-SCT consolidation post-CAR T, and 5/7 died from PD, including 2/5 who died from infection in the context of PD following subsequent post-CAR T treatments.

DISCUSSION

As the clinical demand for CAR T products continues to rise exponentially, optimization of manufacturing workflow to improve product quality, reduce manufacturing complexity, and improve patient access becomes increasingly vital.[21,22] Here, we compared two very different manufacturing methodologies in the COBALT phase I academic clinical trial of 4G7CAR T for LBCL. Process-A was a manual, bag-based, IL-2-supplemented manufacturing process, and process-B was a CD4/8-pre-selected, semi-automated, IL-7/IL-15-supplemented Miltenyi CliniMACS Prodigy-based process.

Our initial findings showed that, in contrast to healthy donors, leukapheresis from patients with LBCL can be highly variable in cell composition. 50% of our patients had less than 50% CD3 T cells in their starting material, and monocyte populations were particularly prominent (Figure 2). It has been reported in the literature that this may represent a potential problem for CAR T manufacturing, as monocytes can phagocytose viral vector and CD3/CD28 activation beads, which can ultimately compromise CAR T yields.[5] Other groups have shown that monocytes reduce T cell activation and proliferation in Dynabead-based manufacturing protocols.[23] For this reason and to standardize immune cell composition of leukapheresis starting material between patients, inclusion of a T-cell enrichment step in CAR-T manufacture can be desirable. While we show that cryopreservation of healthy donor and LBCL patient starting material pre-manufacture can enrich for lymphocyte populations, the results were heterogeneous, and a more reproducible T cell enrichment step using immunomagnetic bead-based CD4/CD8 pre-selection was preferred.[24,25]

During GMP-scaled manufacturing, we obtained significantly higher CD3+ and CAR T cell numbers with process-B compared with process-A, despite shorter manufacture duration (8 vs. 9 days) and lower starting cell numbers (80–100 $\times$ 10^6 T cells vs. 250–750 $\times$ 10^6 PBMCs). It is likely that CD4/CD8 pre-selection and minimal transfer and washing steps in process-B permit better T cell expansion *ex vivo*.

It is possible that the cytokines used in process-B (IL-7/IL-15) compared with process-A (IL-2) and/or the different T cell activation reagents (CTS Dynabeads CD3/CD28 vs. TransAct) contributed to the superior expansion observed, although we did not observe any overt effect of IL-2 vs. IL-7/IL-15 on expansion in small-scale validations (Figure S4).

To date, there is an extensive literature on the use of cytokines to support CAR T manufacturing. Several groups have shown that IL-7/IL-15 supplementation *ex vivo* enhances memory T cell phenotypes[26] and is associated with higher proliferation, lower apoptosis, and selective expansion of naive and central memory T cells when compared with IL-2.[27] Testing IL-2 vs. IL-7/IL-15-cultivated CD19CAR T cells in murine models, Zhou et al. showed that, while initial antitumor activity is similar, CAR T cells expanded in IL-7/IL-15 show better tumor control and animal survival over the longer term.[27] A similar profile has also been observed with the dual-targeting CD20/CD19-CAR T product LV20.19, where IL-7/IL-15 vs. IL-2 is associated with greater CAR T polyfunctionality and polyfunctional strength.[28]

On COBALT, it is possible that the use of IL-7/IL-15 vs. IL-2 contributed to the different kinetics observed between process-A and process-B, but it is unlikely to be the sole factor responsible for the 15–36-fold higher expansion seen in process-B validation runs. Rather, the optimized workflow, with upfront T cell selection, cultivation in a minimally disturbed culture vessel (CentriCult unit/Prodigy) without the cell losses associated with manual transfer between different platforms, and product harvest without a requirement for Dynabead removal/de-beading, is likely to underpin the substantially higher CAR T numbers obtained.

By virtue of the lower starting cell numbers required for process-B, we were able to reduce the volume of viral vector used per process 2.5–7.5-fold without compromising transduction efficiency or final CAR T numbers. For our phase 1 studies, the cost of a batch of GMP-grade lentiviral vector is in the order of $500,000-$1M and is one of the most expensive components of CAR T manufacturing.

Figure 3. COBALT study design, recruitment, 4G7CAR T marking and cytokine analysis

(A) COBALT trial schema

(B) Exemplar flow cytometry showing durable, high-level 4G7CAR T persistence in a responding patient treated with a process-B product.

(C) 4G7CAR T engraftment and persistence over time for all patients by flow cytometry, where 4G7CAR T is expressed as a % of CD3+ T cells in the peripheral blood (PB). Patients receiving process-A products are represented by black lines, and patients receiving process-B products are represented by red lines.

(D) Comparison of peak 4G7CAR T engraftment by flow cytometry between process-A and process-B. Graphs indicate medians. Analysis was carried out using Student's *t* test.

(E) 4G7CAR T engraftment and persistence over time for all patients by transgene-specific qPCR assessment in the peripheral blood (PB). Patients receiving process-A products are represented by black lines, and patients receiving process-B products are represented by red lines.

(F) Comparison of peak CAR engraftment by qPCR between process-A and process-B. Graphs indicate medians. Analysis was carried out by Student's *t* test ($p = 0.1642$).

(G) Peripheral blood (PB) IL-6 and IFN-γ concentrations for individual patients over time to day 28; red = process-B; black = process-A.

Table 4. Summary table of immunotoxicity, responses, relapse, and the nature of relapses on the COBALT study

Maximum grade CRS (UPenn Criteria[20])	
CRS (any)	4/9 (44%)
Grade 2	2/9 (22%)
≥ Grade 3	0/9
Maximum grade Neurotoxicity (CTCAE v4.03)	
Any Grade[a]	5/9 (56%)
Grade 2	1/9 (11%)
Grade 3	0/9
Cytopenias ongoing beyond day 28	
≥ Grade 3 Neutropenia	2/9 (22%)
≥ Grade 3 Thrombocytopenia	1/9 (11%)
Responses	
ORR at Month 1	7/9 (78%)
CMR	3/9 (33%)
PR	4/9 (44%)
SD/PD	2/9 (22%)
Disease Progression/Relapse	
Relapse, CD19+	2/9 (22%)
Relapse, CD19-	2/9 (22%)

[a]Neurological side effects captured by CTCAE v4.03 system included grade 1 headache in 4/9 cases, with onset on day 0 in 2/4, day 3 in ¼, and day 5 in 1/4. The grade 2 neurotoxicity event was lethargy in 1/9 patients, with onset on day 13.

Approaches to reduce vector use per process presents a potentially large cost saving.

From a practical perspective, we observed a substantial reduction in the burden of manufacturing on clean room staff and facilities using process-B. Per product, process-B was less "resource intense" due to the 2.5–7.5-fold lower viral vector usage per process, the 3–4-fold lower grade A clean room time per process (29 vs. 8 h), and the 2-fold lower hands-on operator hours per process (35 vs. 18 h). A substantial cost associated with CAR T manufacturing is the use of GMP clean room space. Process-B has the advantage of reducing the number of open-handling steps which must be performed in a grade A environment within a grade B background. Higher grade environments are associated with elevated running costs due to higher energy demand,[26] as well as extended requirements for gowning, cleaning, and monitoring. The use of a closed-processing approach allows the use of a lower grade clean room (grades C and D) and, importantly, increased parallel processing capacity, therefore diluting fixed running costs. This may represent a significant saving, particularly in a decentralized production model: the literature estimates this to be in the order of 55.7% for 10 batches/year vs. 30.4% for 36 batches/year.[29] Furthermore, higher grade A occupancy and staff hands-on time per product means lower throughput and lower overall manufacturing capacity, which is especially pertinent for small academic facilities with manufacturing responsibility for multiple trials.

On the COBALT study, 10 patient products were manufactured and QP released but in line with differences in expansion observed in the head-to-head analysis using surplus LBCL leucapheresis, only 2/5 products on process-A, including products at the lowest dose level in the study (DL1), and 4/5 on process-B met the target dose. Data from other groups suggest that poor T cell expansion and longer doubling time *in vitro* portend poor expansion and response *in vivo*.[30] Slower expansion *in vitro* is often ascribed to impaired intrinsic T cell fitness, but our data show that the manufacturing process is key, such that the same patient leukapheresis starting material that failed to expand on process-A expanded well on process-B. We also showed higher peak 4G7CAR T expansion by flow cytometry and higher peak serum IL-6 in recipients of products manufactured using process-B. This may suggest that the cells expanded better *in vitro* and *in vivo,* with the caveat that more patients on process-B vs. process-A received DL2.

T cell subsets can be heterogeneous between patients and their CAR T products, and it would be valuable to assess the added impact of different T cell activation reagents and cytokines on this inherent variability. Our current data do not permit comparison of T cell subsets expanded under the different activation and cytokine conditions in processes-A and -B, but it is possible that process-B supported expansion of T cell subsets with greater proliferation potential.

The role of dose escalation in CAR T study design divides opinion, as CAR T toxicity and efficacy are not necessarily issues of dose administered, but rather reflections of CAR T expansion *in vivo*,[31] and CAR T expansion is frequently driven by disease burden. Dose escalation on COBALT was difficult to deliver using process-A due to impaired cell expansion *in vitro*. Using process-B, we managed to comfortably dose patients at DL2 and DL3, demonstrating how this method improved feasibility of study delivery as designed. We cannot definitively confirm whether clinical outcomes observed on COBALT were directly related to dose administered or the manufacturing method used due to the small patient numbers.

In summary, our results demonstrate that process-B, comprising T cell enrichment and semi-automated cell processing in IL-7/IL-15, compares favorably with manufacturing process-A, a non-T cell enriched, bag-based, IL-2 culture method, in reaching the target 4G7CAR T dose, with a concomitant reduction in vector use, grade A clean room occupancy, and staff time in manufacturing.

MATERIALS AND METHODS

4G7 CD19 binder/4G7CAR T preclinical development

The COBALT CD19 binder is derived from the 4G7[13] hybridoma. A codon-optimized 4G7 scFv was incorporated into a CAR format with a CD8a spacer and a 41BBζ endodomain, as described previously.[12] The RQR8 sort-suicide gene[16] was cloned in-frame to permit

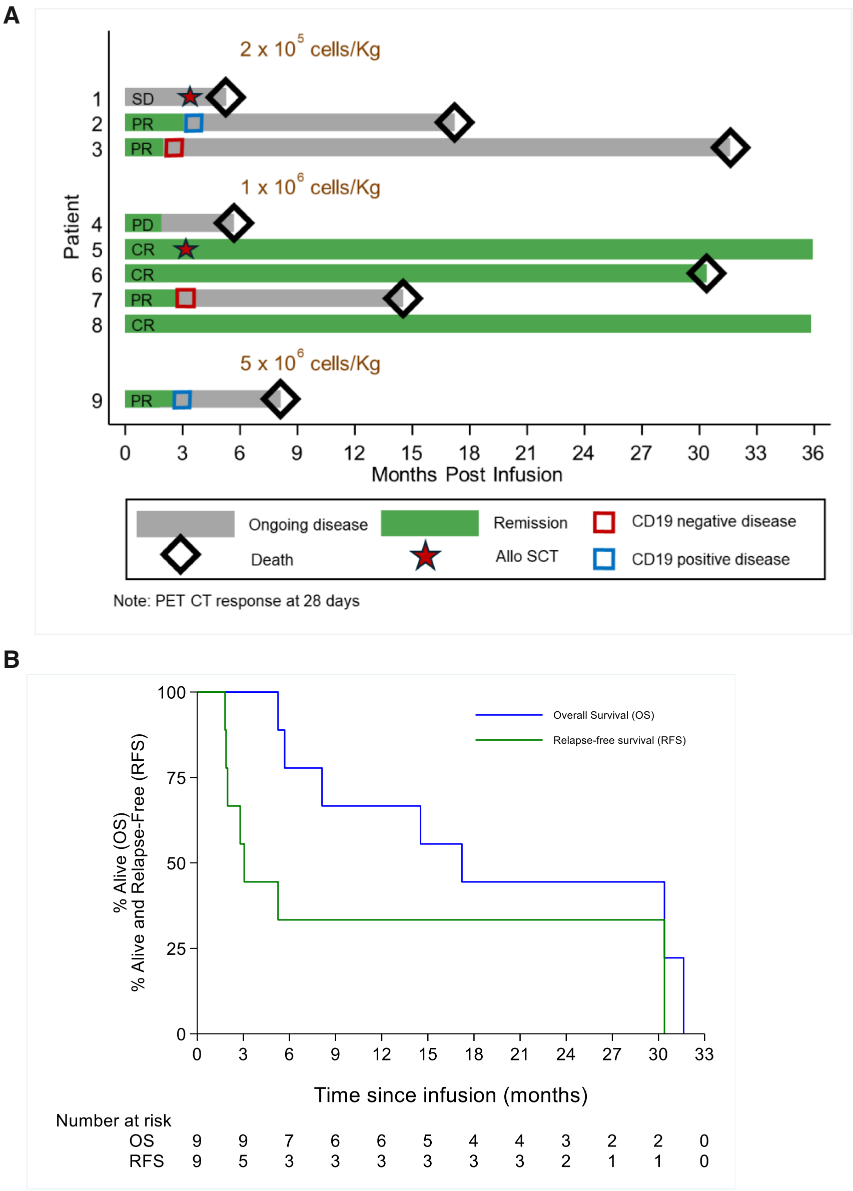

Figure 4. COBALT toxicity and responses

(A) Swimmer's plot of individual patient responses on the COBALT study, subdivided by dose received and manufacture method employed.

(B) Kaplan Meier of OS and PFS for all patients.

deletion of 4G7CAR T using rituximab in the event of toxicity, linked by an FMD-2A like sequence from *Thosea asigna* TaV[32] and expressed in a 3rd-generation self-inactivating (SIN) lentiviral transfer vector, pCCL.RQR8-2A-4g7CAR, with an internal long EF1α promoter. The construct is illustrated in Figure 1A. Clinical grade 3rd-generation SIN lentiviral vector encoding the RQR8-4G7CAR-41BBζ cassette was manufactured to GMP grade, as detailed in the supplemental methods.

4G7CAR T manufacture: Process-A and process-B

Process-A is illustrated in Figure 1B and was initially validated using healthy donor leukapheresis material prior to regulatory submission. Briefly, 5 × 10^8–1 × 10^9 leukapheresis-derived PBMCs were activated with CTS Dynabeads CD3/CD28 on day 0 at a 3:1 cell:bead ratio and cultured in X-VIVO15 supplemented with 5% human AB serum (Seralab) and 120 IU/mL recombinant human IL-2 (Proleukin) until lentiviral transduction on day 2 at an multiplicity of infection (MOI) of 5 in retronectin-coated MACS GMP Cell Differentiation bags (Miltenyi Biotec). On manufacturing day 3, cells were transferred to a WAVE bioreactor for expansion over 4 days, followed by bead removal on day 8 using a CTS DynaMag Magnet and cryopreservation on day 9. For patient products, all steps were performed in a grade A GMP clean room.

Process-B is illustrated in Figure 1B and was initially validated using surplus cryopreserved leukapheresis material from patients with LBCL recruited to COBALT who had already received 4G7CAR T products manufactured by process-A on the clinical study.

Briefly, PBMCs were resuspended in CliniMACS PBS/EDTA (Miltenyi Biotec), supplemented with 1% HAS (Zenalb, Bio Products Laboratory) and loaded onto the CliniMACS Prodigy TS520 tubing set in a grade C clean room prior to CD4/CD8 selection (Miltenyi Biotec), activation with TransAct (Miltenyi Biotec) and cultivation in TexsMACS supplemented with 3% AB serum (Life Science Production) and 10 ng/ml human IL-7 and IL-15 (Miltenyi Biotec) on manufacturing day 0. Lentiviral transduction occurred on manufacturing day 1, followed by cell expansion until manufacturing day 8 and same-day cryopreservation.

GMP clean room time and resources required were estimated per process by reviewing batch manufacturing records (BMRs) for each product and comparing consumables, reagents, equipment, clean room time, and vector use. Release assays are outlined in Table S3.

Study design

COBALT was a single-centre, open-label, dose-escalation phase I study for adult patients (16–65 years) with r/r LBCL failing ≥2 lines of therapy.[33] Inclusion and exclusion criteria are listed in Table S1

alongside the clinical trial protocol. Following non-mobilized leukapheresis and CAR T manufacture, patients received lymphodepletion (LD) with i.v. fludarabine (30 mg per m^2, days −5 to −1) and cyclophosphamide (60 mg/kg, day −7 and day −6) prior to a single dose of 4G7CAR T. DL1 was 2×10^5 4G7CAR T/kg; DL2 was 1×10^6 4G7CAR T/kg, and DL3 was 5×10^6 4G7CAR T/kg (Figure 3A). Where protocol-stipulated 4G7CAR T doses were met, dose escalation continued until three patients in a cohort achieved CMR, in the absence of DLT. Primary endpoints were safety, feasibility of adequate leukapheresis and 4G7CAR T manufacture, and complete response. Endpoints are listed in Table S2.

The study was approved by the UK Medicines and Healthcare Products Regulatory Agency (clinical trial authorization no. 20363/0356/001-0001), the London –West London & GTAC Research Ethics Committee (REC ref no. 15/LO/1509), and the research and development department of University College London Hospital. The study was managed by Cancer Research UK and the University College London Cancer Trials Center. Written informed consent was obtained from patients prior to study entry in accordance with the Declaration of Helsinki. This report incorporates data from all participants who received 4G7CAR T on study before November 12, 2020. Data were locked as of January 6, 2025.

Toxicity and response assessment

Adverse events over the first 28 days post-4G7CAR T infusion were graded according to the Common Terminology Criteria for Adverse Events (CTCAE; version 4.03). Adverse events over the first 28 days post-CAR-DLI were graded according to the CTCAE (version 4.03). CRS was graded according to the UPenn criteria[20] and neurotoxicity was graded as per CTCAE v4.03. Disease response was assessed by positron emission tomography-computed tomography (PET-CT) at protocol-defined time points (pre-LD, months 1,2,3,6,9,12,18,24).[34] Further details are listed in the supplemental appendix.

Correlative studies

Peripheral blood (PB) cytokines and 4G7CAR T expansion/persistence by flow cytometry and qPCR were assessed at protocol-specified time points. Rituximab ELISA and Tisagenlecleucel (Tisa-cel) qPCR of the PB were performed pre-LD.

Cell lines

Raji, K562, and human embryonic kidney (HEK) 293T cell lines were obtained from the American Type Culture Collection. SupT1 cells were purchased from the European Collection of Authenticated Cell Cultures and transduced with an SFG vector to express the human CD19 ectodomain (SupT1-CD19), from which single cells were selected by flow cytometry to generate a CD19-high expression cell line.

Flow cytometry

Flow cytometry was performed on the MACSQuant Analyzer 10 (Miltenyi Biotec), or the Fortessa or Celesta platforms (BD Biosciences), and data analysis was performed using FlowJo v 10.8.0 (Tree Star, Inc., Ashland OR) and FACs DIVA 8.0.1. Expression of CAR was detected by Phycoerythrin (PE)-conjugated anti-murine-F(ab')$_2$ (AffiniPure F(ab')$_2$ Fragment Goat Anti-Mouse IgG, F(ab')$_2$ fragment specific, 115-116-072, Jackson ImmunoResearch), and antigen-presenting cell (APC)-conjugated QBend10 (anti-hCD34 antibody, R & D Systems) to detect RQR8. The following reagents were used for phenotypic analysis of CAR T cells: Anti-CD45 fluorescein isothiocyanate (FITC) clone 2D1 (BD Biosciences), anti-CD3 APC-Cy 7 clone UCHT1 (Biolegend), 7-amino actinomycin D (7-AAD, Miltenyi), goat anti-murine F(ab')2 PE (Jackson ImmunoResearch), anti-CD34 clone QBend10 (R & D Systems), anti-CD8 APC clone SK1 (Biolegend), anti-CD4 VioGreen clone VIT4 (Miltenyi), anti-CD56 (NCAM) BV510 clone HCD56 (Biolegend), anti-CD16 VioGreen clone REA423 (Miltenyi), anti-CD14 APC clone TÜK4 (Miltenyi) or anti-CD14 APC clone 63D (Biolegent), anti-CD279 (PD-1) BV421 clone EH12.2H7 (Biolegend), anti-CD366 (Tim 3) BV510 clone F38-2E2 (Biolegend), anti-CD4 FITC clone OKT4 (Biolegend), anti-CD45RA FITC clone HI100 (eBiosciences), anti-CD197 (CCR7) APC clone G043H7 (Biolegend), anti-CD19 APC clone HIB19 (Biolegend), and Fixable Viability Dye eFluor 780 (eBioscience). Untransduced controls and fluorescence minus one (FMO) controls were used to determine expression thresholds where required.

Preclinical experiments and CAR functionality *in vitro*

Lentiviral supernatants were generated by co-transfection of 293T packaging cells with 3rd-generation lentiviral packaging plasmids pMDLg/pRRE (4.06 μg), RSV-rev (3.13 μg), pMD.G2 (2.92 μg), and the pCCL.RQR8-2A-4G7CAR transfer vector (3.13 μg), using GeneJuice (Merck Millipore). PBMCs were isolated by Ficoll density centrifugation of healthy donor blood on an ethically approved study protocol. Human T cells were transduced on RetroNectin-coated, non-tissue culture-treated 6-well plates following overnight activation with either CTS Dynabead CD3/CD28 (Thermo Fisher Scientific) at a 3:1 bead:cell ratio, or TransAct at 1×10^6 cells/ml at MOIs ranging between 1 and 10. Chromium release and flow-cytometry-based killing assays were performed against GFP-expressing SupT1 non-transduced (NT) and SupT1-CD19 expressing cells. Natural killer cell depletion was achieved using CD56 immunomagnetic beads (Miltenyi Biotec), and 4-h 51-chromium-release cytotoxicity assays were performed against CD19-expressing target cell lines.[35] Percent lysis was calculated as: *% Lysis = (experimental lysis - spontaneous lysis)/(maximum lysis - spontaneous lysis) × 100.* For chromium release and flow-cytometry-based killing assays, effector and target cells were co-cultured at varying ratios for 24 and 48 h. Cultures were stained with PE-conjugated anti-CD3 antibody clone UCHT1 (Biolegend) and Fixable Viability Dye eFluor 780 (eBioscience). CountBright beads (Invitrogen) were used to determine absolute target cells numbers. Effector cells were identified as CD3+GFP- cells, and target cells were identified as CD3-GFP+ (SupT1-NT, SupT1-CD19, and Raji cells). CD19-specific cytotoxicity was determined by the number of live target cells remaining following co-culture with CAR T cells, normalized to the number of live targets in co-cultures with NT T cells.

Coculture assays for cytokine secretion were carried out at a 1:1 target:effector ratio between CAR T cells and eGFP-expressing Raji cell line for 1–7 days. Cytokines were assessed from supernatants obtained from the 24-h 1:1 effector:target ratio co-culture using the CBA Human Th1/Th2 cytokine kit (BD), performed in triplicate and analyzed using the FCAP Array software (Softflow, Inc.).

Clinical lentiviral vector manufacture

A 3rd-generation SIN lentiviral vector encoding the RQR8-2A-4G7CAR-41BBζ cassette under the control of a human EF1α promoter and incorporating the HIV central polypurine tract (cPPT), Rev response element (RRE), and truncated woodchuck hepatitis virus post-transcriptional regulatory element (ΔWPRE) was manufactured for COBALT in accordance with European Medicines Agency (EMA) Guidelines on the Development and Manufacture of Lentiviral Vectors (CHMP/BWP/2458/03) at the Rayne Cell Therapy Suite (RCTS) at King's College London. The lentiviral vector was generated by transient transfection of HEK293T cells using calcium phosphate with plasmids encoding CAR (pCCL), VSVg envelope (pMD.G), HIV gagpol (pMDLg/RRE), and Rev (RSV-Rev). Supernatant subsequently purified using anion exchange chromatography and high-speed centrifugation.

Release assays

Release assays performed prior to infusion included assessments of sterility (Gram stain, bacterial culture, mycoplasma PCR), endotoxin levels by the Limulus Amebocyte Lysate (LAL) method, residual bead count, viability, and transduction efficiency by flow cytometry using RQR8 staining with the anti-hCD34 antibody clone QBend10 (R&D Systems). The material was separately tested for viral copy number.

Correlative studies

Serum cytokine measurements were assessed on days −6, 0, 3, 8, 10, 13, 20, and 28 post-4G7CAR T infusion by cytometric bead array analysis of IL-2, IL-4, IL-6, IL-10, TNF-α, and IFN-γ (BD Biosciences) according to the manufacturer's protocols. Data were analyzed using FCAP Array (Softflow, Inc.). The validated lower limit of this assay is 20 pg/mL. CAR T cell expansion and persistence were assessed in the PB on days −6, 0, 3, 8, 10, 13, 20, 28, and then monthly up to 6 months, followed by every 3 months up to 1 year post-infusion. 4G7CAR T cells were detected using a validated qPCR assay targeting a transgene-specific sequence. Genomic DNA was isolated, and sequencing reactions were carried out with transgene-specific primers and Taqman probes (Applied Biosystems), using a minimum of 0.25 μg genomic DNA where possible. A control qPCR assay using primers and probes for albumin was carried out in parallel to allow calculation of the actual DNA present per sample. Results were reported as copies of the transgene per μg genomic DNA, with a detection limit of 100 copies per μg DNA. Trucount (BD Biosciences) evaluation of absolute T cell numbers (viable, CD45+CD3+ cells) was combined with assessment of the percentage of circulating CAR+CD45+CD3+ T cells (PE-conjugated anti-murine-F(ab')$_2$, Jackson ImmunoResearch) to determine the absolute number of circulating 4G7CAR T cells. RQR8 expression was also assessed by staining with QBend10-APC (R&D Systems). NT PBMCs were used as negative controls.

Tisagenlecleucel qPCR

PB was collected at a single time point (day −6) from all trial patients and centrifuged at 1,700 rcf (g) for 10 min at 4°C. Plasma was separated and stored in aliquots at −80°C. Tisagenlecleucel was detected using a validated qPCR assay targeting a viral packaging signal-specific sequence. Genomic DNA was isolated, and sequencing reactions carried out with transgene-specific primers and Taqman probes (Applied Biosystems), using a minimum of 0.25 μg genomic DNA where possible. A control qPCR assay using primers and probes for albumin was carried out in parallel to calculate the actual DNA present per sample. Results were reported as copies of the transgene per μg genomic DNA.

Toxicity evaluations

Adverse events over the first 28 days post-CAR-DLI were graded according to the CTCAE (version 4.03). CRS was graded according to the UPenn criteria,[20] and neurotoxicity was assessed as per CTCAE v4.03.

Statistical analysis

Only descriptive statistics were employed in the analysis. Continuous variables were summarized using the median and range, where applicable, while categorical variables were reported as frequencies and percentages. Data visualizations, such as plots, were utilized where appropriate. OS was defined as the time from infusion of 4G7CAR T to death from any cause. Relapse-free survival (RFS) was measured as the time from infusion of 4G7CAR T to either relapse or death from any cause, whichever occurred first. RFS events of interest included cases of no response or relapse prior to maintaining a response for at least 28 days. Patients who were censored were those last seen alive, with censoring at the last known date of follow-up. Kaplan–Meier curves were generated to illustrate OS and RFS. Time-to-event outcomes were analyzed using the Kaplan–Meier method, with median OS and RFS times reported, along with OS and RFS rates at 12 and 24 months. Response rates post-infusion were calculated and presented with exact binomial 95% CIs. Swimmer plots were used to depict the duration of remission. Toxicity events were categorized and reported at the maximum grade experienced, while adverse events were summarized by system organ class and CTCAE version 4.03 terms, based on the worst grade reported during the study. Data analysis and visualization were conducted using Stata version 18.5 and GraphPad Prism version 9.

DATA AND CODE AVAILABILITY

For original data, please contact c.roddie@ucl.ac.uk.

ACKNOWLEDGMENTS

This study was supported by a Bloodwise Research grant (ref. 14051). C.R. and K.S.P. were supported by the University College London NIHR Biomedical Research Center (BRC), by the UCL NIHR Blood and Transplant Research Unit (BTRU) in Stem Cells and Immunotherapy at UCL in partnership with the NHS Blood and Transplant

Research Unit, and by core funding through the CRUK London Center. The Cancer Trials Center was supported by a CRUK core grant.

We acknowledge Martin Pule at the UCL Cancer Institute for designing the 4G7 CAR construct. We thank Farzin Farzaneh and Lucas Chan for GMP viral vector manufacture. Work in the Molecular Medicine Group at King's is supported by CRUK, ECMC, and the NIHR BRC based at King's Health Partners.

We acknowledge Kim Champion, Toyin Adedayo, and Nadjet El-Mehidi, who assisted with study documentation. Krystle Villanueva and Fatima Seray-Wurie contributed to clinical trial monitoring and David Gear contributed to data management.

We acknowledge the UCL ECMC GCLP Facility (University College London Experimental Cancer Medicine Center Good Clinical Laboratory Practice Facility, UCL Cancer Institute) for clinical trial endpoint sample analysis, including Victoria Spanswick and Helen Lowe. The UCL ECMC GCLP Facility is funded and supported by ECMC Award C12125/A25143 and the CRUK City of London Application, reference CTRQQR-2021/100004.

We acknowledge the CAR T manufacture personnel at the Institute for Child Health (ICH) at UCL and at the CCGTT (Center for Cell Gene & Tissue Therapeutics) including Hong Zhan, Alison Niewiarowska, Attia Hussain, Kimberly Gilmour, Stuart Adams, Sara Ghorashian, Barry Flutter, Talia Gileadi, Mellisa Cheung, Ines Pereira, Andrei Pobsichan, Rachel Richardson, Nikolaos Gkitsas, Sarah Albon, Paulina Nowosiad, Louisa Green, Mhairi Vaughan, Vitoria Meyer, Rita Rego, and Owen Bain. We also thank Giulia Agliardi and John Garcia for reviewing the manuscript.

At UCLH, contributions from Leigh Wood, Chloe Marden, Clemency Every-Clayton, Louise Enfield, Nivetha Balasubramaniam, Ifrah Aaden, Kathleen Cheok, Strachan MacKenzie, Kasia Jalowiec, and Lorna Neill were invaluable in clinical care and trial conduct, and Miriam Zegeye-Dixon assisted with collection of clinical data. We thank David Linch for his assistance with clinical study management.

We thank Claire Harrison (Guy's and St Thomas' Hospital, London), John Moppett (University Hospital, Bristol), David Miles (Mount Vernon Cancer Center), Paul Silcocks (University of Liverpool), and Caroline Kelly (CR UK Clinical Trials Unit, Glasgow) for providing study oversight as the Independent Data Monitoring Committee. The authors extend their gratitude to all patients and their families, participating sites, and their staff for their support of the study.

AUTHOR CONTRIBUTIONS

K.S.P. and C.R. conceived the study, supervised the project, performed the analysis, and wrote the manuscript. K.S.P., C.R., M.A.O.R., and M.A.V.M. treated patients and/or acquired clinical samples and data. J.D., M.A., A.C.-G., K.V., L.B.-C., M.W.L., G.W.-K.C., N.H., and F.S. manufactured CAR-DLI products. G.W.-K.C., M.M., V.M., and H.R. did preclinical and translational sample analyses. J.A.H., N.M., L.E., and Y.P. conducted GCLP CAR marking and cytokine analysis at the UCL ECMC GCLP laboratory. Trial delivery, management, and statistical analysis was conducted by the UCL CRUK CTC team, including B.P., A.L., and A.D. The vector was supplied by F.F. at KCL. All authors edited and critically reviewed the manuscript.

DECLARATION OF INTERESTS

Since completion of this work, primary affiliations have changed for the following authors: M.A., School of Life Sciences, University College London; A.C.-G., USC/CHLA Cell Therapy Program, University of Southern California, and Children's Hospital of Los Angeles, Los Angeles; K.V., Autolous Therapeutics; L.B.-C., Gene Vector Laboratory, Kings College London; V.M., AstraZeneca; F.F., ViroCell Biologics, Biochemical Engineering Department, University College London; F.S., King Faisal Specialist Hospital and Research Center.

K.S.P. is a shareholder and consultant for Achilles and Autolus Therapeutics. M.W.L. is a consultant to Autolus Ltd. F.F. is affiliated with ViroCell Biologics; holds stock im Autolus Therapeutics, Dawn Therapeutics, and ViroCell Biologics Ltd; and and provides consulting for Autolous Therapeutics and Dawn Therapeutics. C.R. has received speaker fees, honoraria, and advisory board payments from Kite/Gilead, J + J, Autolus, Abbvie, and BMS/Cellistic. M.A.V.M. has received travel support from Takeda. M.A.O.R. has received honoraria from Kite, Novartis, and Janssen and serves om advisory boards for Kite and Autolus. J.A.H. holds stock and has consulting, advisory roles, patents, and royalties with ADC Therapeutics.

SUPPLEMENTAL INFORMATION

Supplemental information can be found online at https://doi.org/10.1016/j.omta.2026.201695.

REFERENCES

1. Schuster, S.J., Svoboda, J., Chong, E.A., Nasta, S.D., Mato, A.R., Anak, Ö., Brogdon, J.L., Pruteanu-Malinici, I., Bhoj, V., Landsburg, D., et al. (2017). Chimeric Antigen Receptor T Cells in Refractory B-Cell Lymphomas. N. Engl. J. Med. *377*, 2545–2554. https://doi.org/10.1056/NEJMoa1708566.
2. Neelapu, S.S., Locke, F.L., Bartlett, N.L., Lekakis, L.J., Miklos, D.B., Jacobson, C.A., Braunschweig, I., Oluwole, O.O., Siddiqi, T., Lin, Y., et al. (2017). Axicabtagene Ciloleucel CAR T-Cell Therapy in Refractory Large B-Cell Lymphoma. N. Engl. J. Med. *377*, 2531–2544. https://doi.org/10.1056/NEJMoa1707447.
3. Abramson, J.S., Palomba, M.L., Gordon, L.I., Lunning, M.A., Wang, M., Arnason, J., Mehta, A., Purev, E., Maloney, D.G., Andreadis, C., et al. (2020). Lisocabtagene maraleucel for patients with relapsed or refractory large B-cell lymphomas (TRANSCEND NHL 001): a multicentre seamless design study. Lancet *396*, 839–852. https://doi.org/10.1016/S0140-6736(20)31366-0.
4. Roddie, C., O'Reilly, M., Dias Alves Pinto, J., Vispute, K., and Lowdell, M. (2019). Manufacturing chimeric antigen receptor T cells: issues and challenges. Cytotherapy *21*, 327–340. https://doi.org/10.1016/j.jcyt.2018.11.009.
5. Stroncek, D.F., Ren, J., Lee, D.W., Tran, M., Frodigh, S.E., Sabatino, M., Khuu, H., Merchant, M.S., and Mackall, C.L. (2016). Myeloid cells in peripheral blood mononuclear cell concentrates inhibit the expansion of chimeric antigen receptor T cells. Cytotherapy *18*, 893–901. https://doi.org/10.1016/j.jcyt.2016.04.003.
6. Stroncek, D.F., Lee, D.W., Ren, J., Sabatino, M., Highfill, S., Khuu, H., Shah, N.N., Kaplan, R.N., Fry, T.J., and Mackall, C.L. (2017). Elutriated lymphocytes for manufacturing chimeric antigen receptor T cells. J. Transl. Med. *15*, 59. https://doi.org/10.1186/s12967-017-1160-5.
7. Ghassemi, S., Nunez-Cruz, S., O'Connor, R.S., Fraietta, J.A., Patel, P.R., Scholler, J., Barrett, D.M., Lundh, S.M., Davis, M.M., Bedoya, F., et al. (2018). Reducing Ex Vivo Culture Improves the Antileukemic Activity of Chimeric Antigen Receptor (CAR) T Cells. Cancer Immunol. Res. *6*, 1100–1109. https://doi.org/10.1158/2326-6066.CIR-17-0405.
8. Sabatino, M., Hu, J., Sommariva, M., Gautam, S., Fellowes, V., Hocker, J.D., Dougherty, S., Qin, H., Klebanoff, C.A., Fry, T.J., et al. (2016). Generation of clinical-grade CD19-specific CAR-modified CD8+ memory stem cells for the treatment of human B-cell malignancies. Blood *128*, 519–528. https://doi.org/10.1182/blood-2015-11-683847.
9. Klebanoff, C.A., Gattinoni, L., Torabi-Parizi, P., Kerstann, K., Cardones, A.R., Finkelstein, S.E., Palmer, D.C., Antony, P.A., Hwang, S.T., Rosenberg, S.A., et al. (2005). Central memory self/tumor-reactive CD8+ T cells confer superior antitumor immunity compared with effector memory T cells. Proc. Natl. Acad. Sci. USA *102*, 9571–9576. https://doi.org/10.1073/pnas.0503726102.
10. Kaiser, A.D., Assenmacher, M., Schröder, B., Meyer, M., Orentas, R., Bethke, U., and Dropulic, B. (2015). Towards a commercial process for the manufacture of genetically modified T cells for therapy. Cancer Gene Ther. *22*, 72–78. https://doi.org/10.1038/cgt.2014.78.
11. Mock, U., Nickolay, L., Philip, B., Cheung, G.W.-K., Zhan, H., Johnston, I.C.D., Kaiser, A.D., Peggs, K., Pule, M., Thrasher, A.J., and Qasim, W. (2016). Automated manufacturing of chimeric antigen receptor T cells for adoptive immunotherapy using CliniMACS prodigy. Cytotherapy *18*, 1002–1011. https://doi.org/10.1016/j.jcyt.2016.05.009.
12. Roddie, C., Dias, J., O'Reilly, M.A., Abbasian, M., Cadinanos-Garai, A., Vispute, K., Bosshard-Carter, L., Mitsikakou, M., Charalambous, E., Mehra, V., et al. (2025). Matched donor allogeneic CAR-T for adult B-ALL: toxicity, efficacy, repeat dosing, and the importance of lymphodepletion. Blood *146*, 1664–1676. https://doi.org/10.1182/blood.2025028790.

13. Meeker, T.C., Miller, R.A., Link, M.P., Bindl, J., Warnke, R., and Levy, R. (1984). A Unique Human B Lymphocyte Antigen Defined by a Monoclonal Antibody. Hybridoma *3*, 305–320. https://doi.org/10.1089/hyb.1984.3.305.

14. Xu, Y., Zhang, M., Ramos, C.A., Durett, A., Liu, E., Dakhova, O., Liu, H., Creighton, C.J., Gee, A.P., Heslop, H.E., et al. (2014). Closely related T-memory stem cells correlate with in vivo expansion of CAR.CD19-T cells and are preserved by IL-7 and IL-15. Blood *123*, 3750–3759. https://doi.org/10.1182/blood-2014-01-552174.

15. Cieri, N., Camisa, B., Cocchiarella, F., Forcato, M., Oliveira, G., Provasi, E., Bondanza, A., Bordignon, C., Peccatori, J., Ciceri, F., et al. (2013). IL-7 and IL-15 instruct the generation of human memory stem T cells from naive precursors. Blood *121*, 573–584. https://doi.org/10.1182/blood-2012-05-431718.

16. Philip, B., Kokalaki, E., Mekkaoui, L., Thomas, S., Straathof, K., Flutter, B., Marin, V., Marafioti, T., Chakraverty, R., Linch, D., et al. (2014). A highly compact epitope-based marker/suicide gene for easier and safer T-cell therapy. Blood *124*, 1277–1287. https://doi.org/10.1182/blood-2014-01-545020.

17. Nastoupil, L.J., Jain, M.D., Feng, L., Spiegel, J.Y., Ghobadi, A., Lin, Y., Dahiya, S., Lunning, M., Lekakis, L., Reagan, P., et al. (2020). Standard-of-Care Axicabtagene Ciloleucel for Relapsed or Refractory Large B-Cell Lymphoma: Results From the US Lymphoma CAR T Consortium. J. Clin. Orthod. *38*, 3119–3128. https://doi.org/10.1200/JCO.19.02104.

18. Kuhnl, A., Roddie, C., Martinez-Cibrian, N., Menne, T.F., Linton, K., Lugthart, S., Chaganti, S., Sanderson, R., Marzolini, M.A.V., Norman, J., et al. (2019). Real-World Data of High-Grade Lymphoma Patients Treated with CD19 CAR-T in England. Blood *134*, 767. https://doi.org/10.1182/blood-2019-124177.

19. Iacoboni, G., Navarro, V., Martín-López, A.Á., Rejeski, K., Kwon, M., Jalowiec, K.A., Amat, P., Reguera-Ortega, J.L., Gallur, L., Blumenberg, V., et al. (2024). Recent Bendamustine Treatment Before Apheresis Has a Negative Impact on Outcomes in Patients With Large B-Cell Lymphoma Receiving Chimeric Antigen Receptor T-Cell Therapy. J. Clin. Orthod. *42*, 205–217. https://doi.org/10.1200/JCO.23.01097.

20. Porter, D., Frey, N., Wood, P.A., Weng, Y., and Grupp, S.A. (2018). Grading of cytokine release syndrome associated with the CAR T cell therapy tisagenlecleucel. J. Hematol. Oncol. *11*, 35. https://doi.org/10.1186/s13045-018-0571-y.

21. Agliardi, G., Dias, J., Rampotas, A., Garcia, J., and Roddie, C. (2025). Accelerating and optimising CAR T-cell manufacture to deliver better patient products. Lancet Haematol. *12*, e57–e67. https://doi.org/10.1016/S2352-3026(24)00273-4.

22. Castella, M., Boronat, A., Martín-Ibáñez, R., Rodríguez, V., Suñé, G., Caballero, M., Marzal, B., Pérez-Amill, L., Martín-Antonio, B., Castaño, J., et al. (2019). Development of a Novel Anti-CD19 Chimeric Antigen Receptor: A Paradigm for an Affordable CAR T Cell Production at Academic Institutions. Mol. Ther. Methods Clin. Dev. *12*, 134–144. https://doi.org/10.1016/j.omtm.2018.11.010.

23. Noaks, E., Peticone, C., Kotsopoulou, E., and Bracewell, D.G. (2021). Enriching leukapheresis improves T cell activation and transduction efficiency during CAR T processing. Mol. Ther. Methods Clin. Dev. *20*, 675–687. https://doi.org/10.1016/j.omtm.2021.02.002.

24. Turtle, C.J., Hanafi, L.-A., Berger, C., Gooley, T.A., Cherian, S., Hudecek, M., Sommermeyer, D., Melville, K., Pender, B., Budiarto, T.M., et al. (2016). CD19 CAR-T cells of defined CD4+:CD8+ composition in adult B cell ALL patients. J. Clin. Investig. *126*, 2123–2138. https://doi.org/10.1172/JCI85309.

25. Rouce, R.H., and Heslop, H.E. (2017). Equal opportunity CAR T cells. Blood *129*, 3275–3277. https://doi.org/10.1182/blood-2017-04-779983.

26. Gattinoni, L., Klebanoff, C.A., and Restifo, N.P. (2012). Paths to stemness: building the ultimate antitumour T cell. Nat. Rev. Cancer *12*, 671–684. https://doi.org/10.1038/nrc3322.

27. Zhou, J., Jin, L., Wang, F., Zhang, Y., Liu, B., and Zhao, T. (2019). Chimeric antigen receptor T (CAR-T) cells expanded with IL-7/IL-15 mediate superior antitumor effects. Protein Cell *10*, 764–769. https://doi.org/10.1007/s13238-019-0643-y.

28. Zurko, J.C., Xu, H., Chaney, K., Fenske, T.S., Hamadani, M., Schneider, D., Hari, P., Johnson, B., and Shah, N.N. (2021). Bispecific LV20.19 CAR T-Cells Expanded in IL-7 and IL-15 Have Greater Polyfunctionality and Polyfunctional Strength Than CAR T-Cells Expanded in IL-2. Blood *138*, 1728. https://doi.org/10.1182/blood-2021-146882.

29. Ran, T., Eichmüller, S.B., Schmidt, P., and Schlander, M. (2020). Cost of decentralized CAR T-cell production in an academic nonprofit setting. Int. J. Cancer *147*, 3438–3445. https://doi.org/10.1002/ijc.33156.

30. Locke, F.L., Rossi, J.M., Neelapu, S.S., Jacobson, C.A., Miklos, D.B., Ghobadi, A., Oluwole, O.O., Reagan, P.M., Lekakis, L.J., Lin, Y., et al. (2020). Tumor burden, inflammation, and product attributes determine outcomes of axicabtagene ciloleucel in large B-cell lymphoma. Blood Adv. *4*, 4898–4911. https://doi.org/10.1182/blood-advances.2020002394.

31. Shah, B.D., Ghobadi, A., Oluwole, O.O., Logan, A.C., Boissel, N., Cassaday, R.D., Leguay, T., Bishop, M.R., Topp, M.S., Tzachanis, D., et al. (2021). KTE-X19 for relapsed or refractory adult B-cell acute lymphoblastic leukaemia: phase 2 results of the single-arm, open-label, multicentre ZUMA-3 study. Lancet *398*, 491–502. https://doi.org/10.1016/S0140-6736(21)01222-8.

32. Donnelly, M.L.L., Hughes, L.E., Luke, G., Mendoza, H., ten Dam, E., Gani, D., and Ryan, M.D. (2001). The “cleavage” activities of foot-and-mouth disease virus 2A site-directed mutants and naturally occurring “2A-like” sequences. J. Gen. Virol. *82*, 1027–1041.

33. Swerdlow, S.H., Campo, E., Pileri, S.A., Harris, N.L., Stein, H., Siebert, R., Advani, R., Ghielmini, M., Salles, G.A., Zelenetz, A.D., and Jaffe, E.S. (2016). The 2016 revision of the World Health Organization classification of lymphoid neoplasms. Blood *127*, 2375–2390. https://doi.org/10.1182/blood-2016-01-643569.

34. Cheson, B.D., Pfistner, B., Juweid, M.E., Gascoyne, R.D., Specht, L., Horning, S.J., Coiffier, B., Fisher, R.I., Hagenbeek, A., Zucca, E., et al. (2007). Revised Response Criteria for Malignant Lymphoma. J. Clin. Oncol. *25*, 579–586.

35. Lugthart, G., Albon, S.J., Ricciardelli, I., Kester, M.G.D., Meij, P., Lankester, A.C., and Amrolia, P.J. (2012). Simultaneous generation of multivirus-specific and regulatory T cells for adoptive immunotherapy. J. Immunother. *35*, 42–53. https://doi.org/10.1097/CJI.0b013e31823569e2.

OMTA, Volume *34*

Supplemental information

A comparison of two manufacturing methods in the phase I COBALT study of CD19CAR T for LBCL

Claire Roddie, Juliana Dias, Gordon Weng-Kit Cheung, Maeve A. O'Reilly, Mahnaz Abbasian, Amaia Cadinanos-Garai, Ketki Vispute, Leticia Bosshard-Carter, Marina Mitsikakou, Vedika Mehra, Harriet Roddy, John A. Hartley, Nasir G. Mahmoud, Leah Ensell, Yashma Patel, Maria A.V. Marzolini, Farzin Farzaneh, Nourredine Himoudi, Farhatullah Syed, Bilyana Popova, Andre Lopes, Alexander Day, Mark W. Lowdell, and Karl S. Peggs

SUPPLEMENTAL TABLES:

Table S1: COBALT inclusion and exclusion criteria

**Inclusion**
• Age 16-65 years<br>• Confirmed diagnosis of CD19+ DLBCL<br>• Primary resistant or relapsed disease failing to achieve metabolic CR to 1st line salvage, or relapse post autograft failing to achieve metabolic CR following a single further cycle of salvage<br>• Potential allogeneic transplant candidate<br>• Agreement to have a pregnancy test, use adequate contraception for 12 months post 4G7CAR-T cell infusion<br>• Karnofsky performance status >60<br>• Written informed consent
**Exclusion at Registration**
• Women who are pregnant or lactating<br>• Prior allogeneic transplantation<br>• Prior history of ischaemic heart disease, dysrhythmias, abnormal ECG, MUGA LVEF<40% (if performed)<br>• Exclusions for proceeding to allogeneic transplantation (active HBV, HCV, HIV; LFT >3 x ULN; CrCl <40 ml/min; or other comorbidity that precludes transplantation)<br>• Known CNS involvement or CVA within prior 3 months<br>• Corticosteroids at a dose of >10mg prednisolone per day (or equivalent)<br>• Rituximab within the last 2 months prior to 4G7CAR-T infusion<br>• Active autoimmune disease requiring immunosuppression<br>• Life expectancy <3 months<br>• Known allergy to albumin or DMSO<br>• Any contraindication to the administration and use of ifosfamide, epirubicin, etoposide, fludarabine and cyclophosphamide.
**Exclusion for CD19CAR T-cell infusion at Day 0**
• Severe intercurrent infection at the time of scheduled 4G7CAR-T cell infusion<br>• Requirement for supplementary oxygen or active pulmonary infiltrates at the time of scheduled 4G7CAR-T cell infusion

Table S2: COBALT primary and secondary endpoints

**Primary endpoints**	**Secondary endpoints**
• Toxicity evaluation following 4G7CAR-T cell administration - most notably attributable AEs and SAEs<br>• Complete tumour response<br>• Feasibility of adequate leukapheresis collection and generation of 4G7CAR-T cells	• Engraftment, expansion and persistence of 4G7CAR-T cells<br>• Depletion of B cell compartment<br>• Timing and magnitude of cytokine release<br>• PET-CT response at 28 days<br>• Number of patients proceeding to allogeneic transplantation

Table S3: COBALT 4G7CAR-T product release criteria

Test/Facility	*Method*	*Specification*
**Cell Count**	FACS: BD Tru- Count™ absolute counting tubes 7AAD, CD45+CD3+ Manual cell count after trypan blue staining	Minimum cell dose<br>1 x 105/kg 4G7CAR-T cells
**Viability of cell product**	BD TruCount™/ Flow Cytometry for CD45, CD3 and 7AAD	≥70% viability (Pre-cryopreservation)
**Transduction efficiency**	Flow Cytometry	≥10% CAR+ cells (Live CD3+/CD45+ T cells)
**Sterility (Bacterial/ Fungal)**	Bacterial cultures	No Growth
**Mycoplasma**	PCR for 16S rRNA	None detected
**Endotoxin**	Kinetic Turbidimetric	≤2EU/ml
**Residual beads**	Manual count	<1bead per 1 x 10^6 cells

Table S4: Detailed demographic information for all registered patients on the COBALT study**.** Key: LD= lymphodepletion; IPI= International Prognostic Index; M/F= male/female; CMR= complete metabolic response; PD= progressive disease; PR= partial response; SD= stable disease; Leuk= leukapheresis (this material was used for head-to-head validations); X, Y, Z are patients with products manufactured on Process-A for the study whose surplus cryopreserved leukapheresis material was used for Process-B validations (see Table 1, main manuscript); *= Product did not meet target dose.

Age (y)	*M/ F*	*Disease*	*IPI*	*Stage pre-LD*	*EN sites*	*Prior Therapy*	*Prior lines*	*Primary refractory*	*Bridging*	*LDH (IU/mL)*
*59	M	DLBCL (GC)	3	4a	Mesentery	CHOP-R x 5 to PD<br>ICE x 2 to PD	3	Yes	Pixantrone x1	481
52 Leuk X	F	tFL (GC)	2	3a	nil	R-CVP x 3 to PR (FL)<br>R-Benda x 6 to CMR + maintenance R (FL)<br>IVE x 2 to SD (tFL)<br>R-GDP x 2 to PD (tFL)<br>Pixantrone to SD (tFL)	5	Yes (once tFL)	Nil	271
55	M	tFL (GC)	1	2a	nil	R-CHOP x 6 + R x 2 to PD<br>R-GDP x 2 to PD	3	Yes	IVE x 1	289
*50 Leuk Y	M	DLBCL (non-GC)	3	4b	Liver	R-CHOP x 8 to PD<br>R-GDP x 1 to PD<br>R-IVE x 1 to PD<br>BTKi + Idelalasib (trial)-stopped due to toxicity<br>Lenalidomide to PD<br>Pixantrone x 1 to PD + mediastinal RT	6	Yes	Nil	292
*52 Leuk Z	M	tFL (GC)	3	4a	Pancreas	R-CHOP x 6 to CMR<br>R-DHAP x 2 to PD<br>IVE x 2 to PR	3	No	Tonsillar RT 30Gy in 15#	317
51	M	CD30+ tFL (GC)	4	4a	Bone marrow	ABVD x 2 to PR (PET+)<br>escBeacopp x 4 to CMR<br>ESHAP x 2 to CMR + LEAM ASCT<br>Brentuximab x 3 to mixed response<br>Nivolumab x 8 to PD	5	No	Steroids	624
63	F	DLBCL (GC)	4	4a	Lung	R-CHOP x 6 (+ HD MTX; IT MTX) to PR + RT to CMR<br>R-GDP x 4 –to mixed response<br>MiniLEAM x 1 to PD<br>Pixantrone x 4 to SD	6	No	IVE x 1	470
44	M	tFL (GC)	3	4a	Pleura	R-CHOP x 6 to CR + maintenance R<br>R-GDP x 2 (complicated by deafness) to PD<br>IVE x 1 to PD	4	Yes	mini-Leam x 1	283
48	M	tFL (GC)	3	4a	Bone	R-CHOP x 6 to PR + IFRT to tonsil to PR<br>R-GDP x 2 to PR<br>IVE x 1 to PR<br>Flu/Cy + Tisagenlecleucel to PD	4	Yes	Steroids	407
*64	F	DLBCL (non-GC)	3	2bx	nil	R-CHOP x 6 to PR<br>R-GDP x 2 to PMR + 30Gy RT in 15# to CMR<br>Flu/Cy + Tisagenlecleucel to PD	4	No	Steroids	537

						RBP x 3 to PR (stopped – cytopenias + infection)				

Table S5: COBALT 4G7CAR-T product details, according to release criteria.

Process	*Fresh/ Frozen Starting Material*	*Target Dose Level (DL)*	*CAR % of CD3*	*CD3 viability %*	*Total CAR T-cell yield (x10^6)*	*Target Dose Met (Yes/No) cryopreserved dose (x10^6)*	*Sterility (no growth 10d)*	*Endotoxin*	*Mycoplasma*
A	Fresh	DL1 2 x 10^5/kg	36.8	95.1	15.8	No (12)	No growth	≤2EU/ml	None detected
A	Fresh	DL1 2 x 10^5/kg	18.1	94.5	13.6	Yes (12.6)	No growth	≤2EU/ml	None detected
A	Fresh	DL1 2 x 10^5/kg	16	94	56.2	Yes (19)	No growth	≤2EU/ml	None detected
A	Fresh	DL2 1 x 10^6/kg	13.7	95	39.7	No (19.8)	No growth	≤2EU/ml	None detected
A	Fresh	DL2 1 x 10^6/kg	15.3	97	64.9	No (58)	No growth	≤2EU/ml	None detected
B	Frozen	DL2 1 x 10^6/kg	35.4	99.7	792	Yes(75.4)	No growth	≤2EU/ml	None detected
B	Fresh	DL2 1 x 10^6/kg	27.9	99.2	529	Yes (91.9)	No growth	≤2EU/ml	None detected
B	Frozen	DL2 1 x 10^6/kg	31.5	99.6	546	Yes (85.5)	No growth	≤2EU/ml	None detected
B	Fresh	DL3 5 x 10^6/kg	19.9	99.5	548	Yes (492)	No growth	≤2EU/ml	None detected
B	Frozen	DL3 5 x 10^6/kg	22.6	99.3	122	No (120)	No growth	≤2EU/ml	None detected

Table S6: Maximum CTCAE grade adverse events observed within 28 days of ATIMP infusion.

Organ Class and AE Name	Maximum AE grade (N=9)			
	1	2	3	4
**Any Adverse Event**				
Any Adverse Event	.	.	.	9 (100%)
**Blood and lymphatic system disorders**				
Any Blood and lymphatic system disorders	.	1 (11%)	8 (89%)	.
Anemia	.	3 (33%)	6 (67%)	.
Febrile neutropenia	.	.	5 (56%)	.
Lymph node pain	1 (11%)	.	.	.
**Cardiac disorders**				
Any Cardiac disorders	1 (11%)	.	.	.
Sinus tachycardia	1 (11%)	.	.	.
**Eye disorders**				
Any Eye disorders	3 (33%)	.	.	.
Dry eye	1 (11%)	.	.	.
Blurred vision	1 (11%)	.	.	.
Vitreous hemorrhage	1 (11%)	.	.	.
**Gastrointestinal disorders**				
Any Gastrointestinal disorders	4 (44%)	.	3 (33%)	.
Nausea	2 (22%)	.	1 (11%)	.
Vomiting	2 (22%)	.	1 (11%)	.
Abdominal pain	1 (11%)	1 (11%)	.	.
Diarrhea	3 (33%)	1 (11%)	1 (11%)	.
Stomach pain	1 (11%)	.	.	.
Anal pain	1 (11%)	.	.	.
Hemorrhoids	1 (11%)	.	.	.
**General disorders and administration site conditions**				
Any General disorders and administration site conditions	3 (33%)	4 (44%)	.	.
Fatigue	3 (33%)	1 (11%)	.	.
Chills	.	1 (11%)	.	.
Fever	1 (11%)	1 (11%)	.	.
Edema limbs	.	1 (11%)	.	.
Neck edema	1 (11%)	.	.	.
**Immune system disorders**				
Any Immune system disorders	2 (22%)	2 (22%)	.	.
Cytokine release syndrome	2 (22%)	2 (22%)	.	.
Other Immune system disorders: Hypogammaglobulinemia	1 (11%)	.	.	.
**Infections and infestations**				
Any Infections and infestations	1 (11%)	3 (33%)	2 (22%)	.
Urinary tract infection	.	.	1 (11%)	.
Tooth infection	.	.	1 (11%)	.
Other Infections and infestations: Unknown	.	1 (11%)	.	.
Other Infections and infestations: Streptococcus oralis	.	1 (11%)	.	.
Lung infection	.	1 (11%)	.	.
Penile infection	.	1 (11%)	.	.
Other Infections and infestations: Streptococcus gordonii	.	.	1 (11%)	.
Other Infections and infestations: Parainfluenza	1 (11%)	.	.	.
Skin infection	.	1 (11%)	.	.

Organ Class and AE Name	Maximum AE grade (N=9)			
	1	2	3	4
**Injury, poisoning and procedural complications**				
Any Injury, poisoning and procedural complications	1 (11%)	.	.	.
Fall	1 (11%)	.	.	.
**Investigations**				
Any Investigations	.	.	.	9 (100%)
Neutrophil count decreased	.	.	.	9 (100%)
White blood cell decreased	.	.	.	7 (78%)
Lymphocyte count decreased	.	.	.	8 (89%)
Platelet count decreased	.	.	1 (11%)	8 (89%)
Lymphocyte count increased	.	1 (11%)	.	.
Alanine aminotransferase increased	1 (11%)	.	.	.
Other Investigations: Raised Ferritin	1 (11%)	.	.	.
Weight gain	1 (11%)	.	.	.
**Metabolism and nutrition disorders**				
Any Metabolism and nutrition disorders	2 (22%)	2 (22%)	.	.
Hypokalemia	1 (11%)	1 (11%)	.	.
Hypophosphatemia	.	2 (22%)	.	.
Anorexia	1 (11%)	.	.	.
Hypocalcemia	1 (11%)	.	.	.
Hyponatremia	1 (11%)	.	.	.
**Musculoskeletal and connective tissue disorders**				
Any Musculoskeletal and connective tissue disorders	2 (22%)	.	.	.
Neck pain	1 (11%)	.	.	.
Joint range of motion decreased	1 (11%)	.	.	.
**Nervous system disorders**				
Any Nervous system disorders	4 (44%)	1 (11%)	.	.
Lethargy	.	1 (11%)	.	.
Headache	4 (44%)	.	.	.
Paresthesia	1 (11%)	.	.	.
Somnolence	1 (11%)	.	.	.
**Psychiatric disorders**				
Any Psychiatric disorders	.	.	1 (11%)	.
Insomnia	.	.	1 (11%)	.
**Renal and urinary disorders**				
Any Renal and urinary disorders	1 (11%)	.	.	.
Urinary tract pain	1 (11%)	.	.	.
**Respiratory, thoracic and mediastinal disorders**				
Any Respiratory, thoracic and mediastinal disorders	4 (44%)	2 (22%)	1 (11%)	.
Cough	4 (44%)	1 (11%)	.	.
Dyspnea	2 (22%)	1 (11%)	.	.
Pharyngeal mucositis	1 (11%)	.	.	.
Pleural effusion	.	.	1 (11%)	.
Laryngeal inflammation	2 (22%)	.	.	.
Sore throat	2 (22%)	.	.	.
**Skin and subcutaneous tissue disorders**				
Any Skin and subcutaneous tissue disorders	3 (33%)	1 (11%)	.	.
Rash maculo-papular	1 (11%)	1 (11%)	.	.
Purpura	1 (11%)	.	.	.
Erythema multiforme	1 (11%)	.	.	.

Table S7: Tabulated results of 6- and 12-month survival rates (all patients). Key: * NE = Not Evaluable; OS (Overall survival). EFS (Event-free survival). OS event = all-cause mortality. EFS event = relapse or all-cause mortality. Median FUP amongst dead patients: 16.1 months [n=7]. Median FUP amongst patients with no dead reported: 32.7 months [n=2]

	OS	***EFS***
Total	n = 9	n = 9
Number of events	7	7
Median time to event in months (95% CI)	17.2 (5.3 to 31.6)	3.1 (1.8 to 30.4)
6 month rate (95% CI)	78% (36% - 94%)	33% (8%-62%)
12 month rate (95% CI)	67% (28% - 88%)	33% (8%-62%)

SUPPLEMENTAL FIGURES

Figure S1

(a)

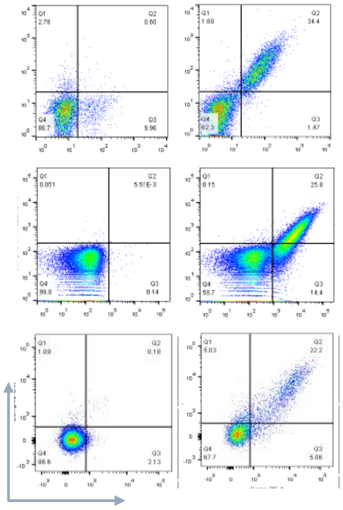

(b)

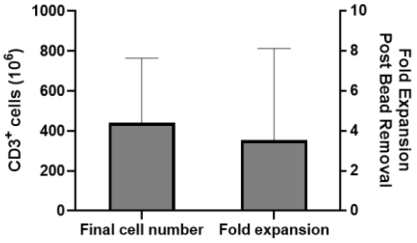

(c)

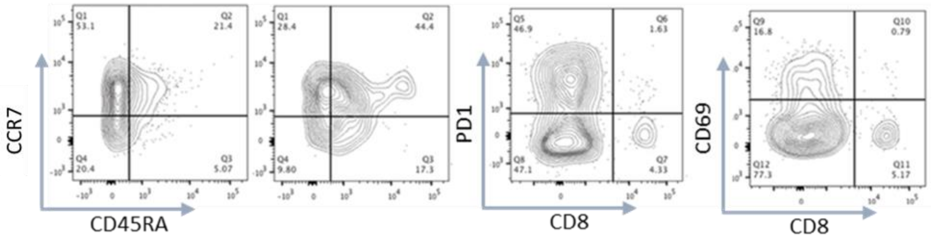

(d)

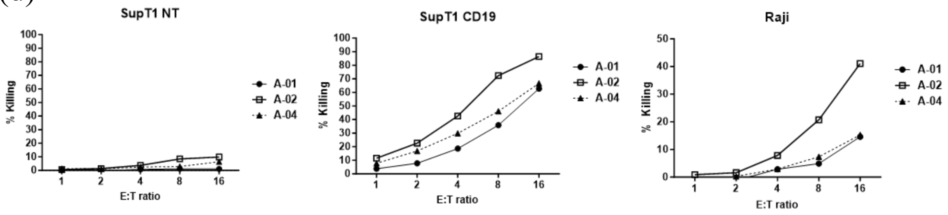

(e)

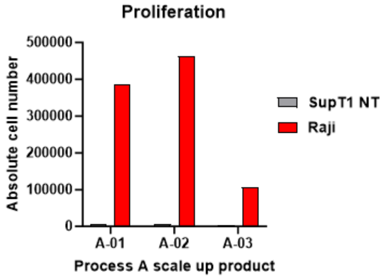

(f)

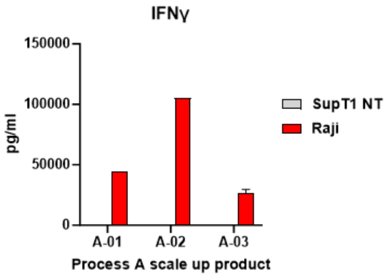

Figure S1: Process A CAR-T product scale-ups using healthy donor PBMCs. Figure S1a: Flow cytometric analysis of scale-up runs. A sample of the cell products compared with NT controls were stained prior to cryopreservation with APC conjugated QBEND/10 and PE-conjugated polyclonal anti-murine-F(ab). The former detects RQR8, while the latter detects the CAR. Cells were analysed by flow-cytometry and fluorescence from QBEND/10 and poly-clonal anti-murine-F(ab) could be independently detected and is shown here as a dot plot with QBEND/10 signal on the y-axis and anti-F(ab) signal on the x-axis. Co-expression of both transgenes is detected. **Figure S1b:** Final cell counts, and fold expansion of the scale-up products. **Figure S1c:** Extended phenotyping of CAR-T cells in an exemplar manufacture process post-thaw using antibodies against CD4, CD8, CCR7, CD45RA, CD69 and PD-1. **Figure S1d:** Chromium release assays were performed against targets: SupT1 NT, SupT1.CD19 and Raji cells at vary effector:target ratios. Post-thaw CAR T-cell products killed SupT1.CD19 and Raji target cells in a dose dependant manner but not SupT1 NT cells. **Figure S1e:** CAR T-cell products proliferate robustly in response to Raji cells following 7-day co-culture. **Figure S1f:** CAR T-cell products produce IFNγ in response to Raji cells in co-culture. Supernatant collected for testing at day 3 of co-culture.

Figure S2

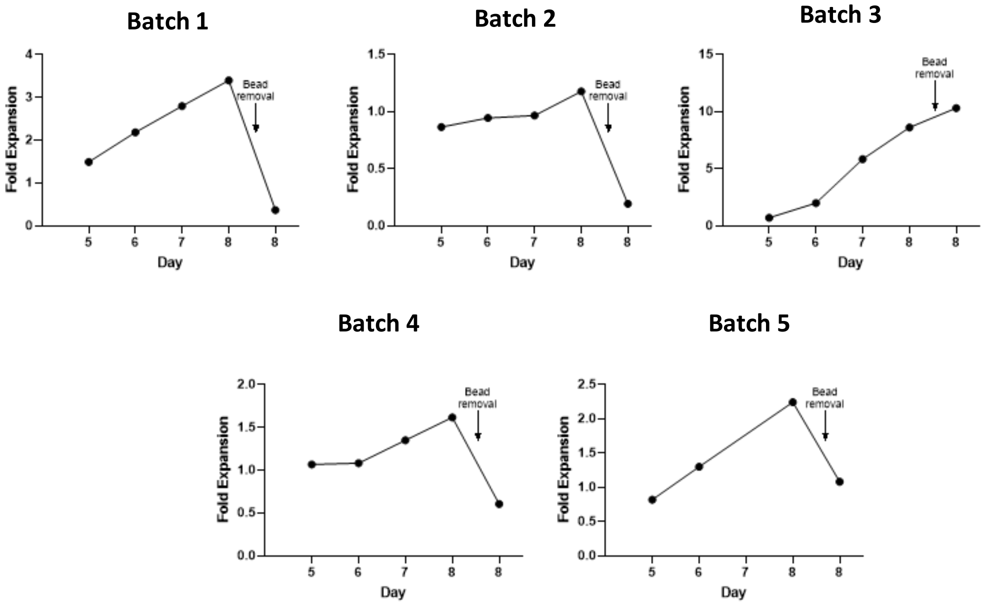

Figure S2: Expansion data for each patient run carried out with Process A. White blood cell (WBC) counts were carried out as in-process controls with the use of a haematological counter (Sysmex). This data reveals the variability of Process A with patient starting material, with a single batch reaching adequate levels of expansion, while 4/5 showed limited cell growth, followed by significant losses during bead removal.

Figure S3

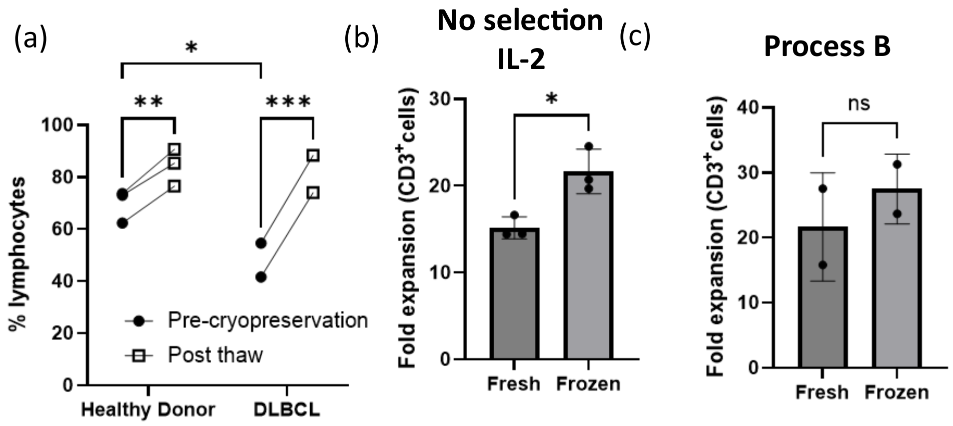

Figure S3: Impact of starting material cryopreservation on manufacturing outcomes. **Figure S3a:** Fold expansion in COBALT batches manufactured using Process B from fresh or frozen patient starting material. Bars represent mean ± SE, Student's t-test, p <0.05. **Figure S3b:** Fold expansion in 4g7 CAR T batches manufactured on the CliniMACS Prodigy using from healthy donors Process B, but with no T cell enrichment step and 100 IU/mL IL-2, instead of 10 ng/mL IL-7/15. Bars represent mean ± SE, Student's t-test, p <0.05. **Figure S3c:** Lymphocyte % obtained from haematological counter (Sysmex) before starting material cryopreservation and or after thaw. LBCL or healthy donor leukapheresis was cryopreserved following standard local procedures. They were thawed in TexMACS medium with 10% human AB serum and rested overnight at 37oC, 5% CO2 without cytokines, before manufacture initiation. Analysis carried out by two-way RM ANOVA

Figure S4

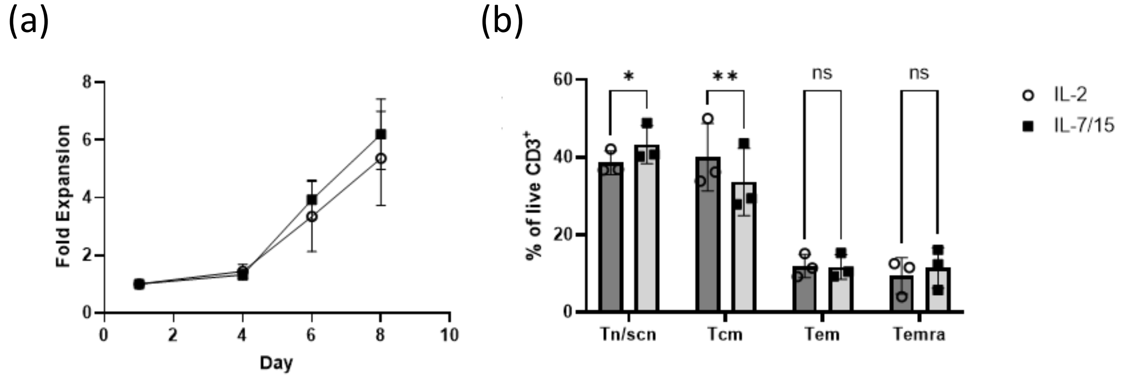

Figure S4: Comparison between IL-2 and a combination of IL-7and IL-15 on T cell expansion and phenotype. Healthy donor cells were obtained from peripheral blood of volunteers. PBMCs were enriched with a Ficoll density gradient and T cells were labelled with the CliniMACS CD4 and CD8 reagents (Miltenyi, 200-070-132 and 200-070-115). They were selected using LS columns (Miltenyi, 130-042-401) with a QuadroMACS™ magnet. Cells were seeded at 1 x 106 CD3+/mL in TexMACS 3% human AB serum and activated using 10 µL/mL human T cell TransAct (Miltenyi, 130-111-160), and 100 IU/mL IL-2 (Miltenyi, 130-097-745) or 10 ng/mL IL-7 (Miltenyi, 130-095-362) and 10 ng/mL IL-15 (Miltenyi, 130-095-764). A culture wash was carried out on day 4, and cells kept at a density of 2 x 106/mL in TexMACS 3% human AB serum and the relevant cytokines, with media exchanges performed every 2 days. **Figure S4a:** T cell expansion in cultures using 100 IU/mL IL-2, or 10 ng/mL IL-7/IL-15. No differences were observed in T cell growth. Plots show represent mean ± SE. **Figure S4b:** Memory phenotype evaluated at the end of culture (day 8). The use of IL-7/15 resulted in a discrete increase in the number of Tn/scm with consequent decrease in Tcm subsets. These results indicate that the combination of IL-7 and IL-15 can maintain the same level of T cell expansion observed with IL-2 while retaining a less differentiated phenotype. Bars represent mean ± SE. Analysis carried out by two-way RM ANOVA with Šídák's multiple comparisons test.

Figure S5

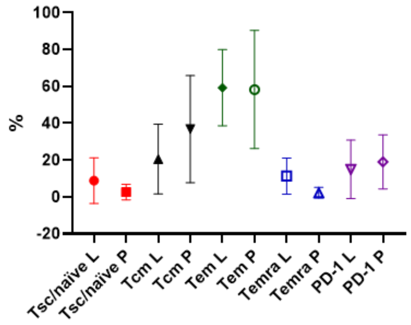

Figure S5: COBALT patient 4G7CAR-T product manufactures. Evolution of memory and exhaustion markers by flow cytometry from leukapheresis starting material (L) to final 4G7CAR-T product (P) on Process-B. T cell subsets defined as previously. Graphs show mean ± SD.

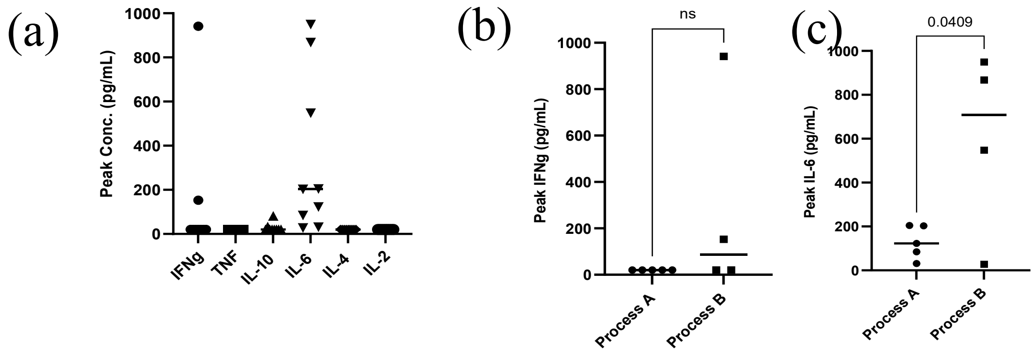

Figure S6: COBALT study cytokine analysis. **Figure S6a:** Summary of peak cytokines for all patients. **Figure S6b:** Comparison of peak IL-6, peak IFNg between Process-A and Process-B.

REFERENCES

1. Ghorashian S, Kramer AM, Onuoha S, Wright G, Bartram J, Richardson R, Albon SJ, Casanovas-Company J, Castro F, *et al.* Enhanced CAR T cell expansion and prolonged persistence in pediatric patients with ALL treated with a low-affinity CD19 CAR. *Nat. Med.* **25**, 1408–1414 (2019).
2. Dull T, Zufferey R, Kelly M, Mandel RJ, Nguyen M, Trono D, Naldini L. A Third-Generation Lentivirus Vector with a Conditional Packaging System. *J. Virol.* **72**, 8463–8471 (1998).
3. Lee DW, Santomasso BD, Locke FL, Ghobadi A, Turtle CJ, Brudno JN, Maus MV, Park JH, Mead E, Pavletic S, *et al.* ASTCT Consensus Grading for Cytokine Release Syndrome and Neurologic Toxicity Associated with Immune Effector Cells. *Biology of Blood and Marrow Transplantation* **25**, 625–638 (2019).
4. MoCA Montreal - Cognitive Assessment. *MoCA Montreal - Cognitive Assessment* https://www.mocatest.org/.
